# Imaging of biphasic signalosomes constructed by checkpoint receptor 2B4 in conventional and chimeric antigen receptor-T cells

**DOI:** 10.1016/j.isci.2024.111669

**Published:** 2024-12-21

**Authors:** Ryohei Matsushima, Ei Wakamatsu, Hiroaki Machiyama, Wataru Nishi, Yosuke Yoshida, Tetsushi Nishikawa, Hiroko Toyota, Masae Furuhata, Hitoshi Nishijima, Arata Takeuchi, Makoto Suzuki, Tadashi Yokosuka

**Affiliations:** 1 Department of Thoracic Surgery, Graduate School of Medical Sciences, Kumamoto University, Kumamoto 860-8556, Japan; 2 Department of Immunology, Tokyo Medical University, Tokyo 160-8402, Japan; 3 Department of Nephrology, Tokyo Medical University, Tokyo 160-8402, Japan; 4 Department of Dermatology, Tokyo Medical University, Tokyo 160-0023, Japan

**Keywords:** Immunology, Cell biology, Methodology in biological sciences

## Abstract

A co-signaling receptor, 2B4, has dual effects in immune cells, but its actual functions in T cells remain elusive. Here, using super-resolution imaging technology with an immunological synapse model, we showed that 2B4 forms “2B4 microclusters” immediately after 2B4–CD48 binding. A lipid phosphatase, SHIP-1, subsequently combined with 2B4 to form coinhibitory signalosomes, leading to the suppression of cytokine production. An activating adapter, SLAM-associated protein (SAP), attenuated the clustering of SHIP-1 and recruited a kinase, Fyn, enhancing the Vav1 signaling pathway as costimulatory signalosomes. Furthermore, we found that a chimeric antigen receptor with a 2B4 tail (2B4-CAR) retained the original signal transduction mechanism of 2B4. With endogenous levels of SAP expression, 2B4-CAR-T cells exposed sufficient antitumor efficacy *in vivo* without excess cytokine production. Our results may help explain the biphasic feature of 2B4 in T cell responses from the viewpoint of the signalosome and provide a new candidate for CAR development.

## Highlights

• 2B4 on T cells forms “2B4 microclusters” after binding to its ligand, CD48
• 2B4 forms coinhibitory signalosomes by translocating SHIP-1 to 2B4 microclusters
• 2B4 forms costimulatory signalosomes by recruiting SAP and Fyn to 2B4 microclusters
• 2B4-CAR prevents the overproduction of inflammatory cytokines *in vitro* and *in vivo*

## Graphical abstract

**Figure:**
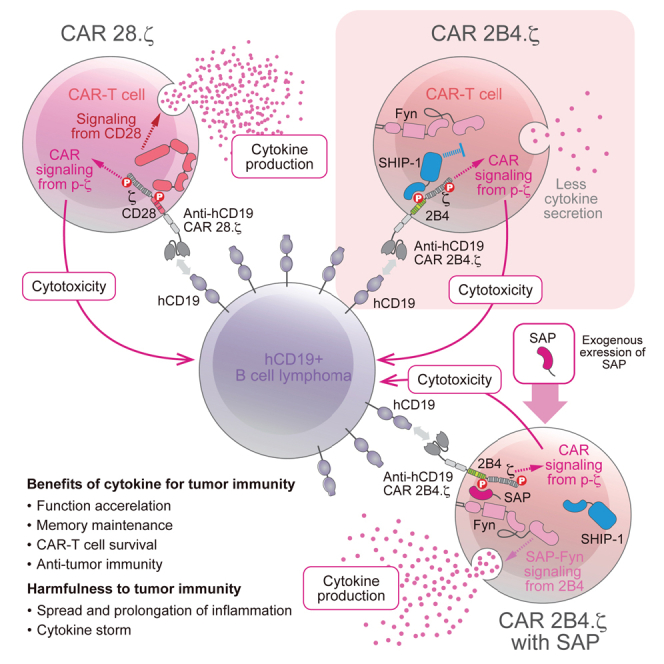

## Introduction

T cell activation requires two distinct signals: one from T cell receptors (TCRs) and the other from various costimulatory or coinhibitory receptors. The signaling lymphocytic activation molecule (SLAM) family receptors, including SLAM, Ly-9, CD84, SLAMF6, and SLAMF7, are known to contribute to immune reactions, mainly as costimulatory receptors broadly expressed on natural killer (NK) cells, T cells, and other hematopoietic cells.^1^ Among the SLAM family receptors, 2B4 (SLAMF4/CD244) (UniProt identifier: human, CD244_HUMAN; mouse, CD244_MOUSE) binds to a ligand, CD48 (human, CD48_HUMAN; mouse, CD48_MOUSE), which is another hematopoietic cell-specific receptor.^2^ 2B4 regulates the activity of NK cells or cytotoxic T cells by binding to CD48 on tumor cells or antigen-presenting cells (APCs). The natural characteristics of multiple myelomas showed therapeutic effects of a monoclonal antibody against CD48, which is highly expressed on plasma cells,^3^ implying that the 2B4–CD48 interaction plays a role in tumor immunity. Interestingly, 2B4–CD48 binding has been demonstrated to lead to both activation and inhibition of T cell responses in various settings.^4^^,^^5^^,^^6^^,^^7^ Thus, 2B4 shows “biphasic functionality,” with both costimulatory and coinhibitory effects on modulating T cell function. Because of that complexity, the confusing mechanisms of how 2B4 regulates T cell function are not well understood or systematized.

2B4 has four immunoreceptor tyrosine-based switch motifs (ITSMs) in its cytoplasmic region.^8^ Phosphorylation of the tyrosine residues in the ITSMs triggered by 2B4–CD48 binding induces the recruitment of several effector molecules containing the Src homology 2 (SH2) domain.^9^ Previous reports have shown that SLAM-associated protein (SAP) (human, SH21A_HUMAN; mouse, SH21A_MOUSE), an activation adapter composed of the SH2 domain, binds to those ITSMs and enhances immune responses in NK cells and T cells.^10^^,^^11^^,^^12^^,^^13^ Congenital mutation or deletion of the *SH2D1A* gene encoding the SAP protein is well known to cause a primary immunodeficiency disease, immunodeficiency condition X-linked lymphoproliferative disease, indicating the biological importance of SAP function in T cell immune responses.^14^^,^^15^ In addition, 2B4 on NK cells associates with several phosphatases such as SH2 domain-containing tyrosine phosphatase (SHP)-1 (human, PTN6_HUMAN; mouse, PTN6_MOUSE), SHP-2 (human, PTN11_HUMAN; mouse, PTN11_MOUSE), and SH2 domain-containing inositol 5′ phosphatase (SHIP)-1 (human, SHIP1_HUMAN; mouse, SHIP1_MOUSE).^11^^,^^16^ In fact, it remains unclear which phosphatase, or all, associates with 2B4 in T cell signaling and function. The 2B4-mediated T cell activation might be determined depending on whether an adapter SAP or any phosphatase is recruited to the ITSMs.^17^^,^^18^^,^^19^ Thus, elucidating the direct linkage between 2B4 signaling and T cell responses may provide some direction for solving clinical issues such as immunodeficiency and tumor immunity.

Chimeric antigen receptor (CAR)-T cell therapy has demonstrated high levels of efficacy against hematologic malignancies. However, it has also challenged physicians and researchers to recognize and manage treatment-associated toxicities. Cytokine release syndrome (CRS), a systemic inflammatory response leading to widespread organ dysfunction, is the most common and severe adverse event associated with CAR-T cell therapies. In clinical trials, the incidence of CRS was between 49% and 95%, with approximately 30% of patients who had undergone CAR-T cell therapy having grade 3 or higher CRS.^20^^,^^21^^,^^22^^,^^23^ Anti-CD19 CAR, in particular, had the highest incidence rate of CRS. In addition to improving the efficacy and therapeutic adaptation of CARs, it is also necessary to develop CARs with fewer treatment-related adverse events.

When a T cell recognizes its cognate antigen peptide in a major histocompatibility complex (MHC) molecule expressed on an APC, an immunological synapse is formed at the adherent interface between the two cells.^24^ Using the combined imaging system of high-resolution microscopy and antigen-presenting supported lipid bilayers (SLBs), our group and others found that a few hundred clusters of TCRs could be imaged at the immunological synapse.^25^^,^^26^^,^^27^ These clusters consisted of tens of TCRs and their proximal signaling molecules. Many of these were tyrosine phosphorylated and have been identified as a minimal unit for T cell activation, named “TCR microclusters.”^25^^,^^26^^,^^27^ The relationship between TCR signaling and the costimulatory effect can be analyzed in terms of microclusters. For example, programmed cell death protein 1 (PD-1), a notable coinhibitory receptor, gathers together in the same regions as TCRs, forming “TCR–PD-1 microclusters,” and associates with SHP-2 to effectively suppress the initial activation of T cells.^28^^,^^29^^,^^30^ Similar to TCRs, tens of CAR molecules were observed to form clusters and transduce downstream signaling as “CAR microclusters” after binding to their ligands.^31^ Visualization of CARs by a single-molecule imaging system combined with SLBs is a useful tool for the spatiotemporal evaluation of the relationship between various signaling molecules and how they affect T cell responses.

Here, we report a T cell engineering imaging analysis that clarified the dynamics of the molecules involved in 2B4 signaling in T cells. Subsequently, we generated a new CAR, 2B4.ζ-CAR, consisting of combined cytoplasm domains of 2B4 and CD3ζ. Our study may provide a deeper understanding of the characteristics of 2B4 and new ideas for the field of CAR development.

## Results

### 2B4 forms microclusters dependently on the binding to CD48

**Figure 1:**
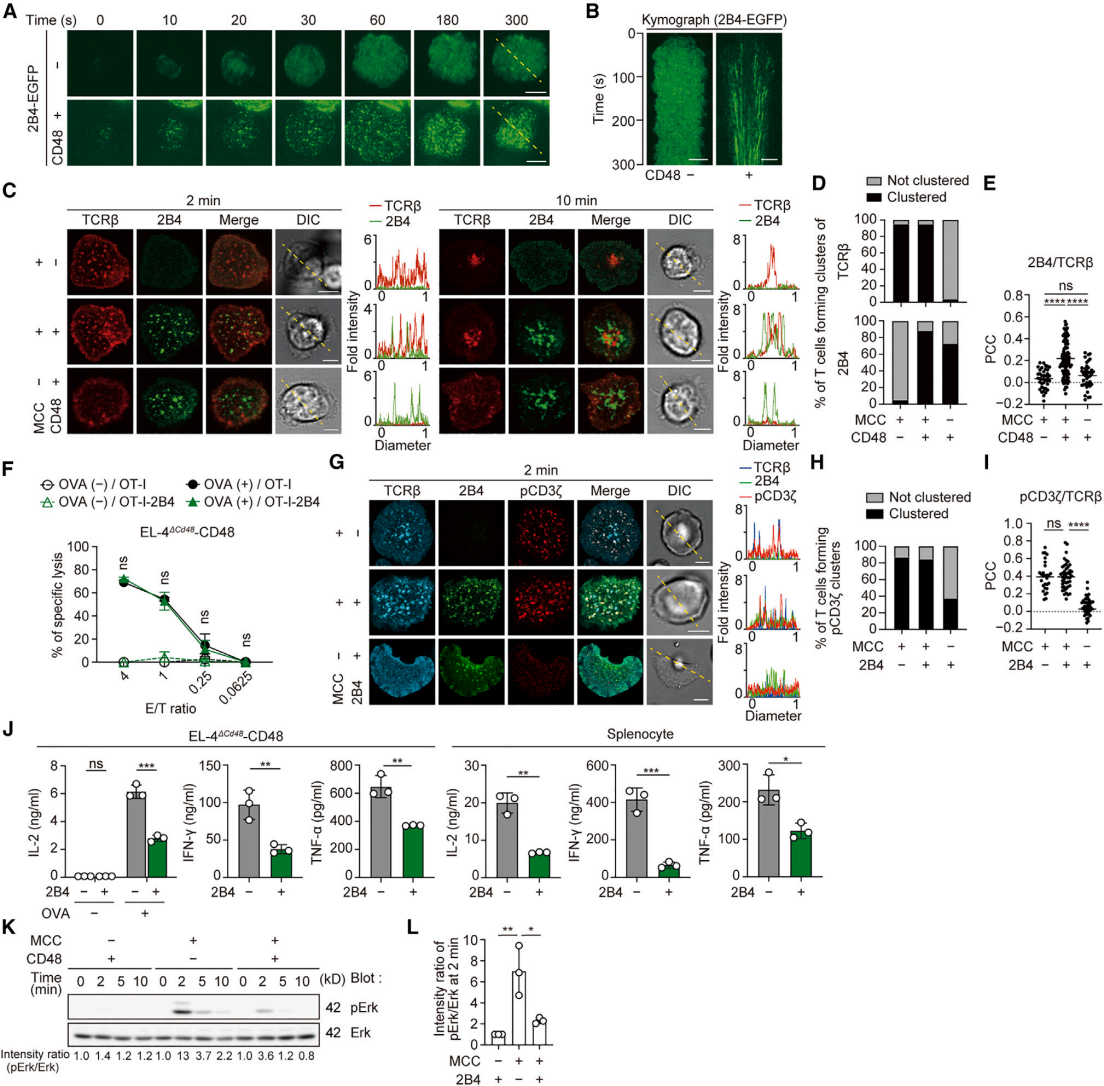
2B4 forms microclusters dependently on the binding to its ligand, CD48 (A) AND-TCR-Tg CD4^+^ T cells expressing m2B4-EGFP were plated onto an MCC_88–103_-prepulsed SLB containing I-E^k^-, mICAM-1-, and without or with mCD48-GPI. A representative of three independent experiments is shown. (B) Clustering and centripetal movement of 2B4 on the diagonal yellow lines in (A) are presented as horizontal elements in kymographs. (C) The cells in (A) were plated onto an MCC_88–103_-prepulsed or not prepulsed SLB without or with mCD48-GPI. Histograms show fold fluorescence intensities of TCRβ and 2B4 on the diagonal yellow lines in the differential interference contrast (DIC) images. A representative of five independent experiments is shown. (D) Percentages of T cells forming TCR or 2B4 microclusters at 2 min in (C) (*n* = 50). (E) Scatterplot summarizing Pearson’s colocalization coefficient (PCC) values at 2 min in (C). PCC was calculated between 2B4/TCRβ by each randomly plotted profile on each cell (MCC_88–103_^+^CD48^−^ [left]; MCC_88–103_^+^CD48^+^ [middle]; MCC_88–103_^−^CD48^+^ [right]; 0.034 ± 0.09, 0.219 ± 0.16, 0.063 ± 0.11; *n* = 40, 101, 40; respectively). (F) OT-I-TCR-Tg effector CD8^+^ T cells non-transduced or transduced with m2B4 were co-cultured for 16 h with target mCD48-deleted EL-4 cells reconstituted by mCD48 (EL-4^*ΔCd48*^-CD48) not prepulsed or prepulsed by 1 μM OVA_257–264_. The percentage of specific lysis with and without 2B4 expression against OVA_257–264_-prepulsed target cells was statistically analyzed. A representative of three independent experiments is shown. (G) 2D12 cells not expressing or expressing m2B4-EGFP were plated onto an MCC_88–103_-prepulsed or not prepulsed SLB with mCD48-GPI. A representative of three independent experiments is shown. (H) The percentages of T cells forming pCD3ζ microclusters in (G) (*n* = 120). (I) The scatterplot summarizes the PCC values between pCD3ζ/TCRβ in (G) (MCC^+^2B4^−^, MCC^+^2B4^+^, MCC^−^2B4^+^ T cells; 0.398 ± 0.16, 0.402 ± 0.19, 0.058 ± 0.09; *n* = 28, 45, 45; respectively). (J) Cytokine secretion assay by ELISA. The T cells in (F) were co-cultured with mCD48-expressing EL-4 cells not prepulsed or prepulsed by 1 μM OVA_257–264_ (left) or with splenocytes prepared from C57BL/6 mice and 1 nM OVA_257–264_ (right) for 6 h. A representative of three independent experiments is shown. (K) 2D12 cells expressing m2B4 were stimulated by mCD48-deleted or mCD48-expressing EL-4 cells not prepulsed or prepulsed by 10 μM MCC_88–103_. A representative of three independent experiments is shown. (L) Each intensity ratio of pErk/Erk at 2 min for the case of MCC^−^2B4^+^ in (K) is plotted on the graph. Bars, 5 μm. Data are presented as mean values ± SD. Statistical analysis was performed using a two-sided *t*-test or one-way ANOVA. ∗*p* < 0.05, ∗∗*p* < 0.01, ∗∗∗*p* < 0.001, ∗∗∗∗*p* < 0.0001. ns, not significant. See also Figure S1.

We first performed precise analysis to localize 2B4 and determine its behavior in T cells using a high-resolution total internal reflection fluorescence (TIRF) microscope with SLBs. SLBs were reconstituted by glycosylphosphatidylinositol (GPI)-anchored murine MHC class II, I-E^k^, and ICAM-1 as basic components, and by the further addition GPI-CD48, a ligand of 2B4. For T cells to be imaged for 2B4, splenic CD4^+^ T cells were freshly prepared from AND-TCR (specific for moth cytochrome *c* 88–103 [MCC_88–103_] on I-E^k^) transgenic (Tg) Rag2-deficient (*Rag2*^*−/−*^) mice and stimulated once for the retroviral transduction of enhanced green fluorescent protein-tagged 2B4 (2B4-EGFP) (Figure S1A). In the presence of CD48, the cells that settled on the SLBs formed clusters of 2B4 at the nascent contact region of the T cell–SLB interface (Figures 1A and 1B, and Video S1). These 2B4 clusters migrated toward the center of the interface, known as the central-supramolecular activation cluster (c-SMAC) of the immunological synapse. To examine the colocalization of 2B4 and TCRs in the presence of both CD48 and MCC_88–103_ peptides on SLBs, AND-Tg T cells expressing 2B4-EGFP were prestained with DyLight 650-labeled anti-TCRβ (H57) Fab and imaged as in Figure 1A. Both 2B4 and TCRβ formed clusters at the initial contact region between T cells and SLBs, and these clusters migrated into the c-SMACs (Figures 1C and 1D). 2B4 microclusters were mostly merged with TCRs and partly adjacent to them. Pearson’s colocalization coefficient (PCC) values between 2B4 and TCR clusters demonstrated moderate averages (Figure 1E) compared with those of other costimulatory or coinhibitory receptors, which we previously reported.^30^^,^^32^ As 2B4-EGFP reconstituted into AND-TCR–expressing CD4^+^ T cell hybridoma (2D12) or OT-I-TCR (specific for ovalbumin 257–264 [OVA_257–264_] on H-2K^b^)–expressing CD8^+^ T cell hybridoma (OT91) showed similar behavior to that in primary T cells (Figures S1A–S1E), we speculated that 2B4 would perform similar functions in any type of cell. To confirm the localization of 2B4 at a T cell–APC interface, CD8^+^ OT-I-TCR T cells expressing 2B4-EGFP were conjugated with EL-4 cells, an H-2K^b+^ lymphoma cell line with CD48 deleted using CRISPR/Cas9 (EL-4^*ΔCd48*^) or reconstituted with CD48 (EL-4^*ΔCd48*^-CD48) (Figure S1F). The accumulation of 2B4 at the T cell–EL-4 cell interface was observed only if CD48 was expressed on EL-4 cells (Figure S1G). These data clearly demonstrated that 2B4 formed clusters with TCRs at the nascent contact region of the T cell–SLB interface upon binding to its ligand, CD48.

Video S1. 2B4 forms microclusters in the presence of CD48 AND-TCR-Tg CD4^+^ T cells transduced with m2B4-EGFP were plated onto an MCC_88–103_-prepulsed SLB containing I-E^k^-, mICAM-1-, and without (left) or with mCD48-GPI (right) and real-time imaged using TIRF microscopy every 2.5 s. Bars, 5 μm. A representative of two independent experiments is shown.

### 2B4 on T cells suppresses cytokine production, but not cytotoxicity, through binding to CD48

**Figure 2:**
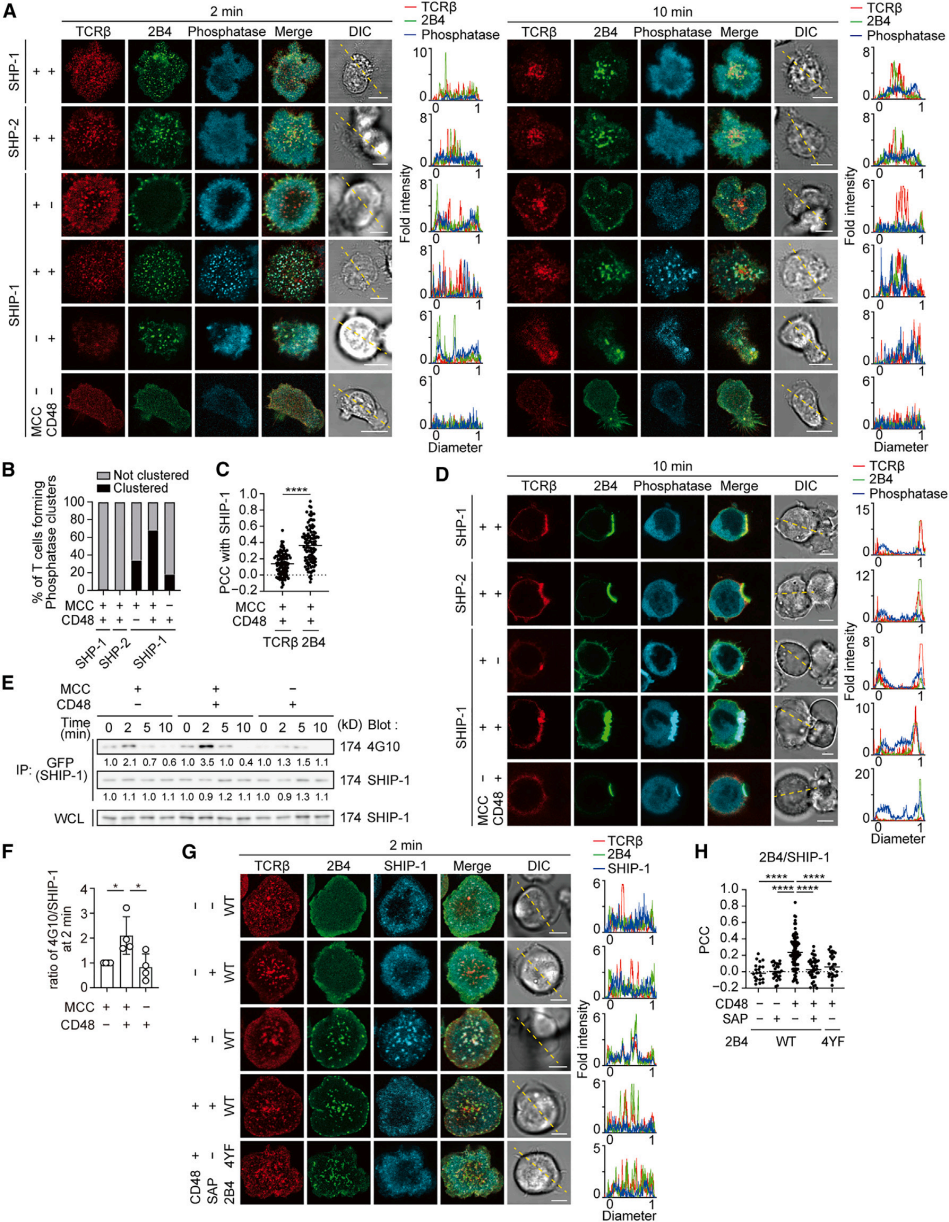
SHIP-1 colocalizes with 2B4 at TCR microcluster depending on the 2B4–CD48 binding (A) AND-TCR-Tg CD4^+^ T cells transduced with 2B4-EGFP and HaloTag-SHP-1, HaloTag-SHP-2 or SHIP-1-HaloTag were plated onto a not prepulsed SLB or MCC_88–103_-prepulsed SLB without or with CD48-GPI. A representative of four independent experiments is shown. (B) Percentages of T cells forming microclusters of each phosphatase at 2 min in (A) (*n* = 50). (C) The PCC values of SHIP-1-expressing T cells plated on the MCC_88–103_-prepulsed SLB with CD48-GPI 2 min after contact in T cells of row 4 in (A). PCC was calculated between SHIP-1/TCRβ (0.138 ± 0.14, *n* = 105) or SHIP-1/2B4 (0.366 ± 0.22, *n* = 105). (D) 2D12 cells expressing 2B4-EGFP and HaloTag-SHP-1, HaloTag-SHP-2, or SHIP-1-HaloTag were conjugated with CD48-deleted or CD48-expressing EL-4 cells not prepulsed or prepulsed by MCC_88–103_. A representative of two independent experiments is shown. (E) 2D12 cells expressing SHIP-1-EGFP and 2B4-HaloTag were stimulated by mCD48-deleted or mCD48-expressing EL-4 cells not prepulsed or prepulsed by 10 μM MCC_88–103_. Lysates were immunoprecipitated for SHIP-1 by anti-GFP. A representative of three independent experiments is shown. (F) Each intensity ratio of 4G10/SHIP-1 (IP) at 2 min for the case of MCC^+^CD48^−^ in (E) is plotted on the graph. (G) 2D12 cells were transduced with SHIP-1-HaloTag and 2B4 (wild type)-EGFP or 2B4-4YF-EGFP and additionally introduced with SAP as indicated. These cells were plated onto an MCC_88–103_-prepulsed SLB without or with CD48-GPI. A representative of three independent experiments is shown. (H) Scatterplot summarizing the PCC values between 2B4/SHIP-1 in (G) (−0.016 ± 0.12, 0.005 ± 0.09, 0.233 ± 0.21, 0.027 ± 0.14, 0.064 ± 0.15; *n* = 22, 26, 80, 40, 28; respectively from the left of the graph).Bars, 5 μm. Data are presented as mean values ± SD. Statistical analysis was performed using two-sided *t*-test or one-way ANOVA. ∗*p* < 0.05, ∗∗∗∗*p* < 0.0001. See also Figure S2.

We next evaluated the functional potency of 2B4 in T cells. We prepared splenic CD8^+^ T cells from OT-I-TCR-Tg *Rag2*^*−/−*^ mice, reconstituted with 2B4, and examined cytotoxicity by co-culture with EL-4 cells expressing CD48 (Figure S1A). Interestingly, CD8^+^ T cells expressing 2B4 showed similar cytotoxicity to T cells without 2B4 expression (Figure 1F). Our previous reports showed that the phosphorylation of CD3ζ was attenuated at the TCR microclusters upon PD-1–PD-L1 binding that suppressed T cell cytotoxicity.^28^^,^^29^^,^^30^ We examined whether the initial signaling via TCR was affected by 2B4–CD48 binding in the same way as it was affected by PD-1–PD-L1 binding. As expected, 2B4–CD48 binding neither attenuated nor enhanced phosphor (p)CD3ζ staining at TCR microclusters (Figure 1G). Quantitative analysis also confirmed that there were no changes in the proportion of the cells forming TCR clusters stained by anti-pCD3ζ and in the ratio of pCD3ζ staining to total TCR (pCD3ζ/TCR) (Figures 1H and 1I). In addition, in the absence of TCR stimulation, although dim staining of pCD3ζ was observed (Figures 1G–1I), 2B4–CD48 interaction alone did not induce any cytotoxic T cell response (Figure 1F). The cytotoxicity against CD48-negative EL-4 cells also did not differ between T cells with or without 2B4 expression (Figure S1H).

We next assessed whether 2B4–CD48 interaction affected cytokine production. 2B4^+^ OT-I T cells stimulated by OVA_257–264_-pulsed CD48^+^ EL-4 cells produced less IL-2, IFN-γ, and TNF-α than 2B4^*−*^ T cells (Figure 1J, left). In the absence of CD48 on EL-4 cells, the production of these cytokines from 2B4^+^ T cells was equal to that from 2B4^*−*^ T cells (Figure S1I). To confirm that 2B4-mediated suppression of cytokine production could be observed in response to the physiological level of CD48 expression on APCs, primary splenocytes (i.e., those expected to simply express endogenous CD48) were prepared from C57BL/6 mice (Figure S1J). We then stimulated 2B4^+^ T cells with these splenocytes and OVA_257–264_ peptides and confirmed lesser production of IL-2, IFN-γ, and TNF-α from T cells in a similar manner to when CD48^+^ EL-4 cells were used as APCs (Figure 1J, right). Western blotting analyses also demonstrated a reduction in the phosphorylation state of Erk, a further downstream molecule of the TCR/CD3 complex (Figures 1K and 1L). These findings suggested that 2B4 potentially possesses some inhibitory mechanism to suppress TCR downstream signaling, resulting in reduced production of various cytokines, but not to affect proximal TCR signaling sufficiently to induce cytotoxicity.

### Src homology 2 domain-containing inositol 5′ phosphatase-1 colocalizes with 2B4 at the T cell receptor microcluster depending on 2B4–CD48 binding

To elucidate the inhibitory molecule involved in the suppression of cytokine production via 2B4-mediated signaling in T cells, we first examined the association of several phosphatases with 2B4, particularly at TCR–2B4 microclusters. AND-Tg T cells transduced with 2B4-EGFP plus HaloTag-tagged phosphatases, SHP-1, SHP-2, or SHIP-1, which have been reported to associate with 2B4 (Figure S2A), were imaged; SHIP-1, but not SHP-1 or SHP-2, was demonstrated to form clusters colocalizing with 2B4 (Figure 2A, left). TCR signaling alone induced the formation of SHIP-1 clusters at TCR microclusters, consistent with previous reports showing the involvement of SHIP-1 in TCR signaling.^33^^,^^34^ However, in the presence of both MCC_88–103_ peptides and CD48 on the SLBs, the formation of SHIP-1 microclusters was enhanced by 2B4–CD48 binding and SHIP-1 microclusters showed higher PCC with 2B4 microclusters than with TCR microclusters (Figures 2B and 2C). In the absence of TCR-MHC peptide binding, the cells did not adhere or spread sufficiently, but the clustering of SHIP-1 could be observed just in the presence of 2B4–CD48 binding. In a later phase of T–SLB conjugation, SHIP-1 was translocated toward the center of the interface where it strongly accumulated, forming a c-SMAC together with 2B4 (Figure 2A, right). This was confirmed by cell–cell conjugation assays, which showed that the accumulation of SHIP-1 at the immunological synapse correlated with 2B4–CD48 binding (Figure 2D). To further understand the involvement of the phosphatases in 2B4, we examined the phosphorylation state of SHIP-1 or SHP-1 in immunoprecipitation experiments, defined previously by Veillette et al., on the relationship between 2B4 and the phosphatase in NK cells.^10^ Tyrosine phosphorylation of SHIP-1 was detected at early time points when T cells expressing both 2B4 and SHIP-1 were stimulated by MCC_88–103_ peptide-prepulsed CD48^*−*^ EL-4 cells (Figures 2E and 2F). As shown in the imaging analysis, however, phosphorylation of SHIP-1 was enhanced by 2B4–CD48 binding and also slightly detected when T cells were stimulated with CD48^+^ EL-4 cells without any cognate peptides. These findings were not observed for SHP-1 (Figure S2B).

Next, to assess how SAP, an SLAM family adapter molecule, affected the behavior of SHIP-1, we simultaneously introduced 2B4, SHIP-1, and SAP into AND-TCR T cells and performed the same imaging analysis. Whereas SHIP-1 microclusters were formed independently on SAP expression in the absence of CD48, co-expression of SHIP-1 with SAP resulted in a reduction of PCC between 2B4 microclusters and SHIP-1 microclusters in the presence of CD48 (Figures 2G and 2H). This suggested that SAP suppressed the 2B4-mediated recruitment of SHIP-1 to TCR–2B4 microclusters. Moreover, T cells expressing mutants of 2B4 containing the substitution of tyrosine with phenylalanine residues in all four ITSMs (2B4-4YF) showed the attenuation of SHIP-1 clustering. Taken together, these results demonstrate that SHIP-1 is involved in the 2B4-mediated inhibitory machinery in T cells collaborating with an adapter SAP.

### 2B4 recruits both signaling lymphocytic activation molecule-associated protein and Fyn at TCR–2B4 microclusters dependently on the binding to CD48

**Figure 3:**
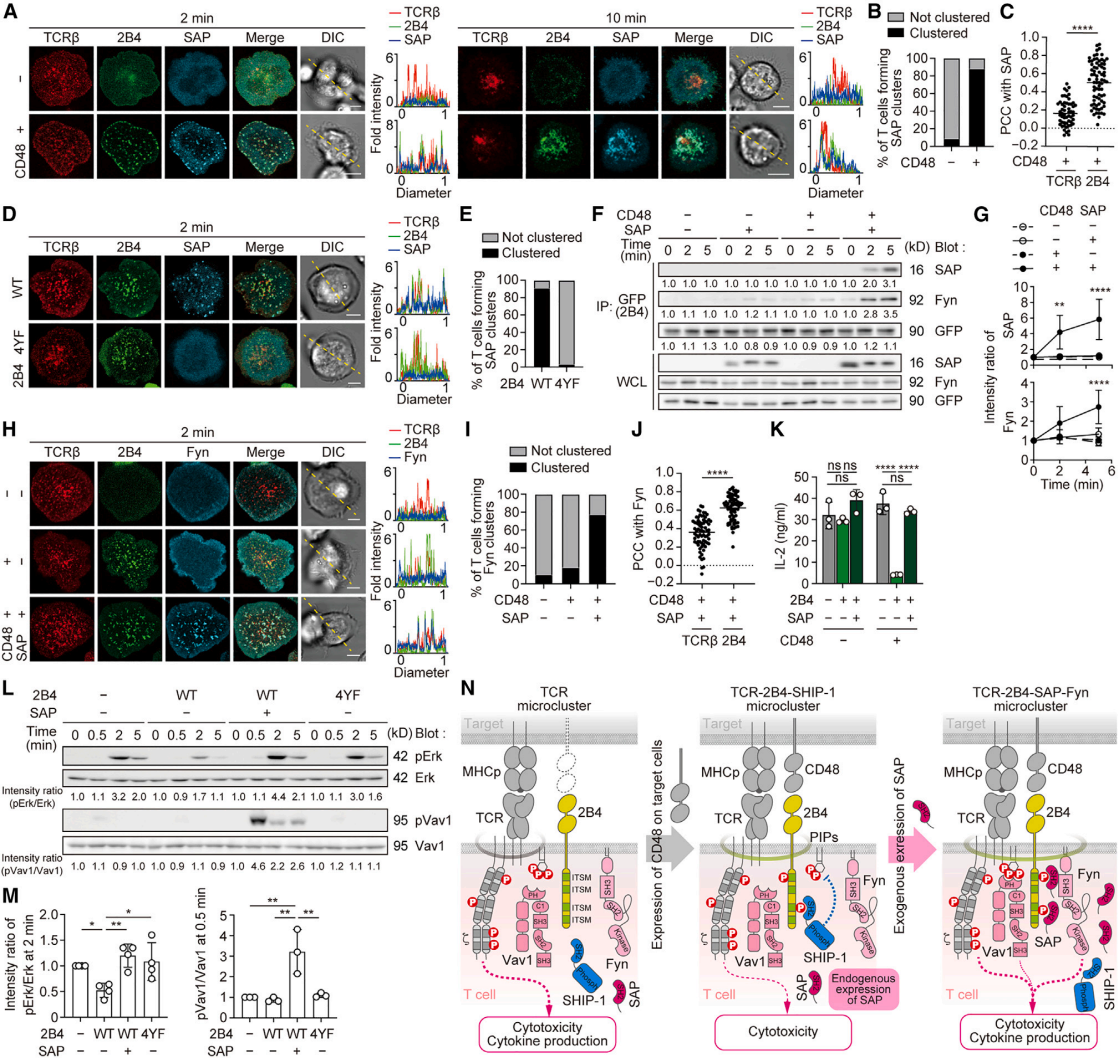
2B4 recruits both SAP and Fyn at TCR-2B4 microclusters depending on binding to CD48 (A) AND-TCR-Tg T cells transduced with 2B4-EGFP and SAP-HaloTag were plated onto an MCC_88–103_-prepulsed SLB without or with CD48-GPI. A representative of four independent experiments is shown. (B) Percentages of T cells forming SAP microclusters at 2 min in (A) (*n* = 70). (C) PCC was calculated between SAP/TCRβ (0.173 ± 0.14, *n* = 55) and SAP/2B4 (0.508 ± 0.23, *n* = 75) in T cells of the left bottom row in (A). (D) 2D12 cells transduced with SAP-HaloTag and wild type (WT) 2B4-EGFP or 2B4-4YF-EGFP were plated onto an MCC_88–103_-prepulsed SLB with CD48-GPI. A representative of two independent experiments is shown. (E) Percentages of T cells forming SAP microclusters in (D) (*n* = 30). (F) 2D12 cells expressing 2B4-EGFP and Fyn-HaloTag were not transduced or transduced with SAP and were stimulated by mCD48-deleted or mCD48-expressing EL-4 cells prepulsed by MCC_88–103_. Lysates were immunoprecipitated for 2B4 by anti-GFP. A representative of three independent experiments is shown. (G) Intensities of SAP or Fyn in (F) are shown. (H) The T cells in (F) were plated onto an MCC_88–103_-prepulsed SLB without or with CD48-GPI. A representative of three independent experiments is shown. (I) Percentages of T cells forming Fyn microclusters in (H) (*n* = 70). (J) PCC was calculated between Fyn/TCRβ (0.360 ± 0.16, *n* = 72) and Fyn/2B4 (0.622 ± 0.14, *n* = 72) in T cells of row 3 in (H). (K) IL-2 secretion assay by ELISA. OT91 cells non-transduced or transduced with 2B4 alone or both 2B4 and SAP were co-cultured with mCD48-deleted or mCD48-expressing EL-4 cells prepulsed with OVA_257–264_. A representative of three independent experiments is shown. (L) 2D12 cells non-transduced or transduced with WT 2B4, 2B4-4YF, or both WT 2B4 and SAP were stimulated by mCD48-expressing EL-4 cells prepulsed by MCC_88–103_. A representative of three independent experiments is shown. (M) Each intensity ratio of pErk/Erk at 2 min or pVav1/Vav1 at 0.5 min for the case of 2B4^−^SAP^−^ in (L) is plotted on the graph. (N) The schematic model of 2B4 signaling pathways without CD48 on target cells (left), with CD48 on target cells and little SAP in T cells (middle), and with CD48 on target cells and sufficient SAP in T cells (right). Bars, 5 μm. Data are presented as mean values ± SD. Statistical analysis was performed by two-sided *t*-test or one-way ANOVA. ∗*p* < 0.05, ∗∗*p* < 0.01, ∗∗∗∗*p* < 0.0001. ns, not significant. See also Figure S3.

We next investigated how SAP, which inhibited the recruitment of SHIP-1 to 2B4, behaved in imaging upon 2B4–CD48 binding. Using AND-Tg T cells co-expressing 2B4-EGFP and SAP-HaloTag (Figure S3A), we demonstrated that, in the presence of CD48, SAP initially colocalized with 2B4, forming microclusters, and sequentially accumulated in a distinct region within a c-SMAC together with 2B4 (Figures 3A–3C). Accumulation of SAP at an immunological synapse was further imaged in the cell–cell conjugation assay (Figure S3B). Meanwhile, 2B4-4YF abolished the formation of the SAP microcluster upon stimulation by SLBs reconstituted with CD48 (Figures 3D and 3E). The physical association of SAP with 2B4 was also detected when 2B4^+^ T cells were stimulated by MCC_88–103_-prepulsed CD48^+^ EL-4 cells (Figures 3F and 3G). We next examined the involvement of Fyn (human, FYN_HUMAN; mouse, FYN_MOUSE), a Src family protein tyrosine kinase, in 2B4. Fyn transduced into 2B4^+^ T cells clustered at 2B4 microclusters depending on SAP expression (Figures S3H-S3J and S3C). We confirmed that the recruitment of Fyn to 2B4 coincided with that of SAP in an immunoprecipitation assay (Figures 3F and 3G).

To examine how these results affect T cell responses, we analyzed the response of 2B4^+^ OT-I-TCR T cells introduced or not introduced by exogenous SAP upon stimulation with OVA_257–264_-pulsed CD48^+^ APCs. We found that the suppression of IL-2 production mediated by 2B4–CD48 binding was restored by the additional transduction of SAP into 2B4^+^ T cells (Figures 3K and S3A). This effect was also seen in 2B4^+^ AND-TCR T cells (Figure S3D). OT-I-TCR T cells expressing both 2B4 and SAP showed a slight enhancement in cytotoxicity at an E/T ratio of 0.25 (Figures S3A and S3E). Western blot analysis demonstrated the increased phosphorylation of both Erk and Vav1 triggered by 2B4-mediated signaling through SAP and Fyn (Figures 3L and 3M). EAT-2 (human, SH21B_HUMAN; mouse, SH21B_MOUSE) is another adapter protein known to directly associate with 2B4 in NK cells.^35^^,^^36^^,^^37^ We examined how EAT-2 behaved in T cells upon 2B4–CD48 binding and found that EAT-2 formed clusters colocalized with 2B4 in a similar fashion as SAP but had no effect on IL-2 production (Figures S3F–S3I). To summarize these data, 2B4 forms coinhibitory signalosomes by the translocation of SHIP-1 into TCR-2B4 microclusters (Figure 3N, middle). Whereas, SAP prevents the forming of SHIP-1 microclusters and translocates Fyn into TCR-2B4 microclusters to generate activating signalosome for further downstream regulation of TCR (Figure 3N, right).

### 2B4.ζ-chimeric antigen receptor forms chimeric antigen receptor microclusters in a CD19 binding–dependent fashion

**Figure 4:**
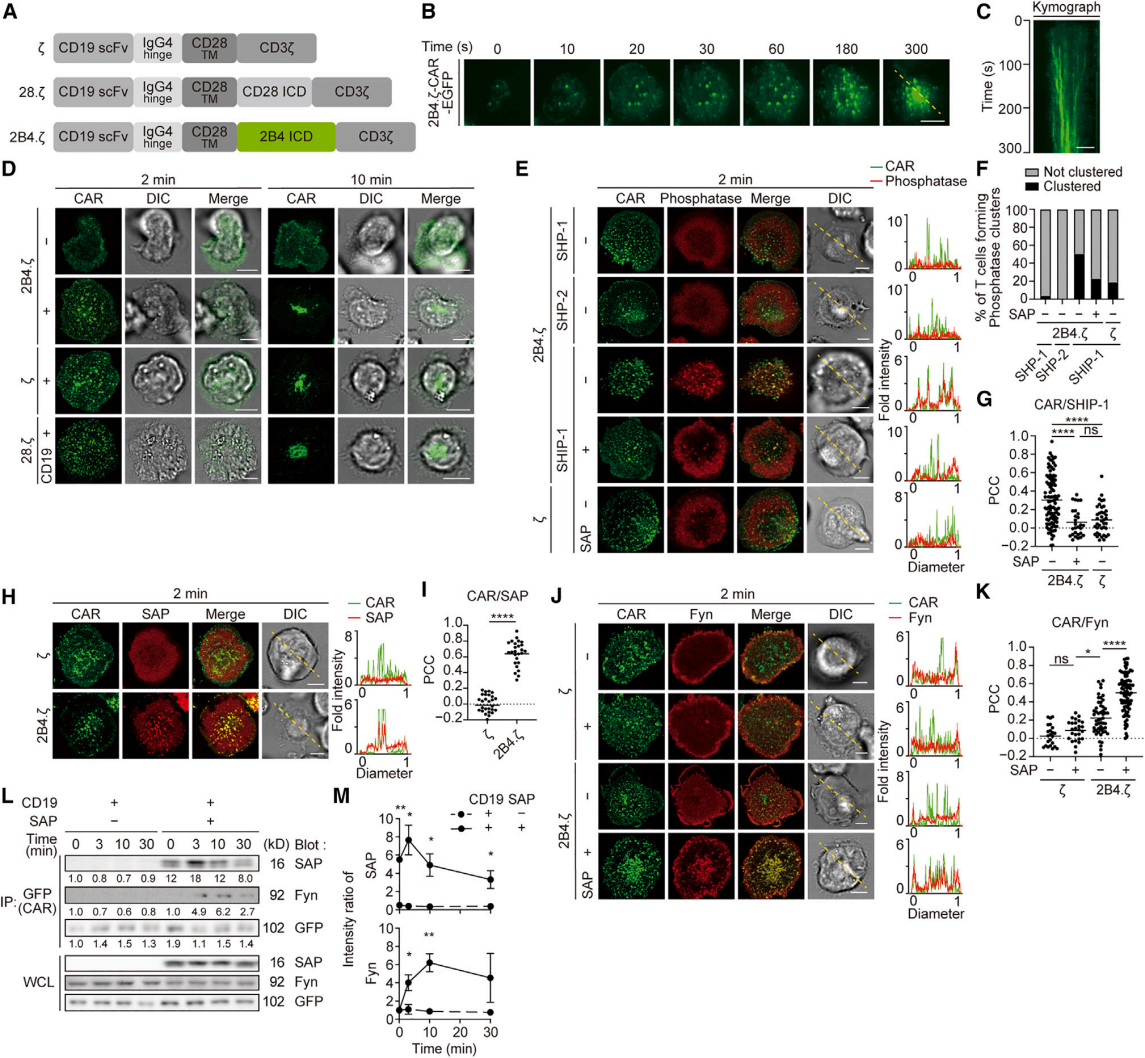
Translocation of SHIP-1 was visualized specifically at 2B4.ζ-CAR microclusters but suppressed by SAP expression (A) Schematics of the CARs composed of anti-hCD19 antibody scFv, hinge of IgG4, transmembrane domain (TM) of CD28, intracellular domains (ICDs) of CD3ζ plus CD28 or 2B4. (B) B6 CD3^+^ T cells transduced with 2B4.ζ-CAR-EGFP were plated onto an SLB containing mICAM-1- and hCD19-GPI. A representative of two independent experiments is shown. (C) Clustering and centripetal movement of 2B4.ζ-CAR on the diagonal yellow lines in (B) are presented as horizontal elements in a kymograph. (D) Splenic T cells were prepared from C56/BL6 mice, transduced with ζ-CAR-EGFP, 28.ζ-CAR-EGFP, or 2B4.ζ-CAR-EGFP, plated onto an SLB with or without CD19-GPI. A representative of four independent experiments is shown. (E) 2D12 cells expressing 2B4.ζ-CAR-EGFP/ζ-CAR-EGFP and HaloTag-SHP-1/HaloTag-SHP-2/SHIP-1-HaloTag were further transduced with SAP and plated onto an SLB containing CD19-GPI. A representative of four independent experiments is shown. (F) Percentages of T cells forming microclusters of each phosphatase in (E) (*n* = 50). (G) PCC was calculated between CAR/SHIP-1 in (E) (0.303 ± 0.27, 0.060 ± 0.15, 0.083 ± 0.16; *n* = 98, 28, 40, respectively, from the left of the graph). (H) 2D12 cells expressing 2B4.ζ-CAR-EGFP or ζ-CAR-EGFP, and SAP-HaloTag were plated onto an SLB containing CD19-GPI. A representative of four independent experiments is shown. (I) PCC values between CAR/SAP in (H) (ζ, 2B4.ζ; −0.019 ± 0.13, 0.644 ± 0.16; *n* = 30, 26, respectively). (J) 2D12 cells expressing Fyn-HaloTag and 2B4.ζ-CAR-EGFP or ζ-CAR-EGFP were introduced with SAP and plated on an SLB containing CD19-GPI. A representative of three independent experiments is shown. (K) PCC values between CAR/Fyn in (J) (0.019 ± 0.13, 0.087 ± 0.11, 0.225 ± 0.19, 0.491 ± 0.21; *n* = 24, 26, 52, 89; respectively from the left of the graph). (L) 2D12 cells expressing 2B4.ζ-CAR-EGFP and Fyn-HaloTag were not transduced or transduced with SAP and were stimulated by EL-4 cells expressing CD19, lysed, immunoprecipitated for CAR by anti-GFP. A representative of three independent experiments is shown. (M) Intensities of SAP or Fyn in (L) are shown. Bars, 5 μm. Data are presented as mean values ± SD. Statistical analysis was performed by two-sided t-test or one-way ANOVA. ∗*p* < 0.05, ∗∗*p* < 0.01, ∗∗∗∗*p* < 0.0001. ns, not significant. See also Figure S4.

CAR-T cell therapy has great potential clinical utility but often causes CRS due to excessive release of inflammatory cytokines. Having understood the characteristics of 2B4 in conventional T cells, we hypothesized that incorporating the cytoplasmic region of 2B4 into CAR might reduce the harm of those cytokines. We generated three CARs derived from a single-chain variable fragment (scFv) that recognizes human (h) CD19, consisting of the hinge of IgG4 and the transmembrane domain of hCD28, which is directly linked to the intracellular signal domains. Each CAR construct contained CD3ζ (ζ), the cytoplasmic region of CD28 plus CD3ζ (28.ζ), or the cytoplasmic region of 2B4 plus CD3ζ (2B4.ζ) as a costimulatory amplifier (Figure 4A). We first imaged the behavior of 2B4.ζ-CAR using TIRF microscopy. Splenic CD3^+^ T cells prepared from C57BL/6 mice were stimulated and then retrovirally transduced by EGFP-tagged 2B4.ζ-CAR (Figure S4A). 2B4.ζ-CAR formed clusters at the nascent contact region between a T cell and an SLB containing CD19 and migrated toward the center of an immunological synapse in the same way shown by TCR and other costimulatory or coinhibitory receptors (Figures 4B and 4C, and Video S2). We also confirmed microcluster formation and centripetal movement, not only in 2B4.ζ-CAR but also in ζ-CAR and 28.ζ-CAR (Figures 4D and S4B). Accumulation of CAR at the CAR-T–EL-4 cell interface was further imaged by cell–cell conjugation assay (Figures S4C and S4D).

Video S2. 2B4.ζ-CAR forms microclusters in the presence of CD19 B6 CD3^+^ T cells transduced with 2B4.ζ-CAR-EGFP were plated onto an SLB containing mICAM-1- and hCD19-GPI, and real-time imaged using TIRF microscopy every 2.5 s. Bars, 5 μm. A representative of two independent experiments is shown.

### Src homology 2 domain-containing inositol 5′ phosphatase-1 specifically accumulates at 2B4.ζ-chimeric antigen receptor microclusters depending on chimeric antigen receptor-CD19 binding

We next examined which phosphatase might contribute to the signaling pathway from 2B4.ζ-CAR. AND-TCR-Tg T cells transduced by 2B4.ζ-CAR-EGFP with HaloTag-tagged SHP-1, SHP-2, or SHIP-1 were conjugated with CD19^+^ EL-4 cells. By imaging, we showed that SHIP-1, but neither SHP-1 nor SHP-2, accumulated at the T cell–EL-4 cell interface (Figure S4E). Similarly, the T cells settled on CD19-reconstituted SLBs showed just SHIP-1 clustering colocalized at 2B4.ζ-CAR microclusters (Figure 4E). Although ζ-CAR also formed SHIP-1 microclusters, 2B4.ζ-CAR showed a higher proportion of T cells forming SHIP-1 microclusters and a higher PCC between CAR and SHIP-1 than did ζ-CAR (Figures 4F and 4G). Furthermore, similar to the results of primary T cells forming 2B4-SAP signalosomes in Figure 2G, co-expression of 2B4.ζ-CAR and SAP reduced the SHIP-1 clustering at CAR microclusters to the same level as ζ-CAR.

We then investigated whether SAP and Fyn contributed to signaling with 2B4.ζ-CAR by forming active signalosomes as well as those of 2B4 in T cells. 2B4.ζ-CAR-T cells, but not ζ-CAR-T cells, showed clustering of SAP at 2B4.ζ-CAR microclusters and formed Fyn clusters if 2B4.ζ-CAR-T cells were additionally introduced by SAP (Figures 4H–4K, S4F, and S4G). The physical association of SAP with Fyn was shown in a biological analysis using T cells expressing 2B4.ζ-CAR stimulated with CD19^+^ EL-4 cells (Figures 4L and 4M). These data suggested that 2B4.ζ-CAR might utilize the original signal transduction pathway of 2B4 by forming a functional signalosome with SAP and Fyn.

### 2B4.ζ-chimeric antigen receptor-T cells demonstrate sufficient cytotoxicity but produce lower amounts of cytokines *in vitro*

**Figure 5:**
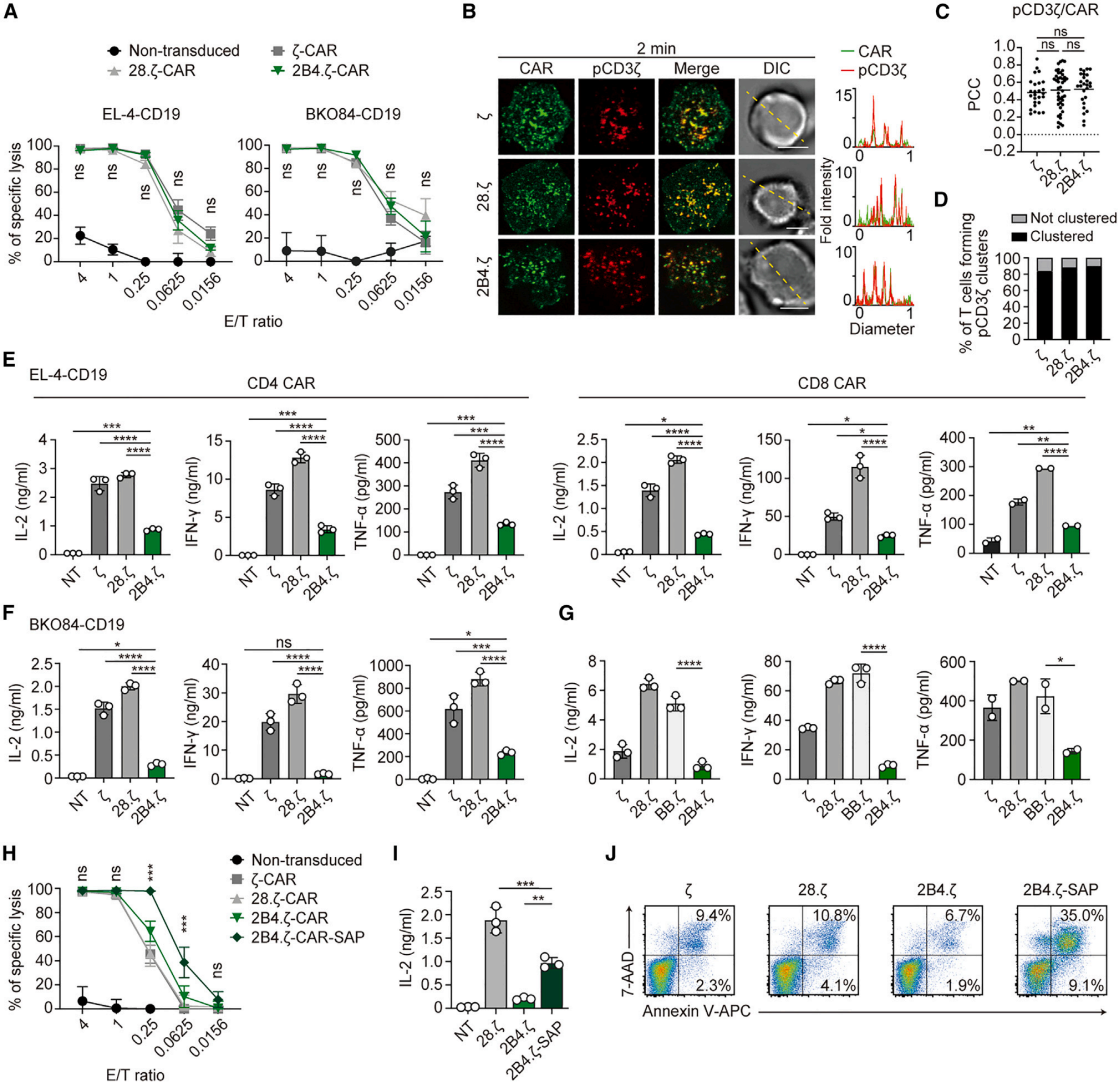
2B4.ζ-CAR-T cells demonstrate equivalent cytotoxicity but produce lower amounts of cytokines compared with other CAR-T cells *in vitro* (A) CD8^+^ T cells from C57BL/6 mice were not transduced or transduced with ζ-CAR, 28.ζ-CAR, or 2B4.ζ-CAR and co-cultured with CD19-expressing EL-4 cells or BKO84 cells at the indicated E/T ratios for 16 h. Statistics were performed for each CAR. A representative of three independent experiments is shown. (B) 2D12 cells expressing ζ-CAR-EGFP, 28.ζ-CAR-EGFP, or 2B4.ζ-CAR-EGFP were plated onto an SLB containing CD19-GPI. A representative of two independent experiments is shown. Bars, 5 μm. (C) PCC values between pCD3ζ/CAR in (B) (ζ, 28.ζ, 2B4.ζ; 0.479 ± 0.17, 0.510 ± 0.22, 0.525 ± 0.19; *n* = 26, 43, 26; respectively). (D) Percentages of T cells forming pCD3ζ microclusters in (B) (*n* = 70). (E) CD4^+^ (left) or CD8^+^ (right) T cells were not transduced (NT) or transduced with ζ-CAR, 28.ζ-CAR, or 2B4.ζ-CAR and co-cultured with EL-4 cells expressing CD19. A representative of three independent experiments is shown. (F) ζ-CAR-, 28.ζ-CAR-, or 2B4.ζ-CAR-T cells were co-cultured with BKO84 cells expressing CD19. A representative of two independent experiments is shown. (G) CD3^+^ T cells were transduced with ζ-CAR, 28.ζ-CAR, 4-1BB.ζ-CAR, or 2B4.ζ-CAR and co-cultured with EL-4 cells expressing CD19. A representative of two independent experiments is shown. (H) CD8^+^ T cells in (A) or 2B4.ζ-CAR-T cells further transduced with SAP were co-cultured with EL-4 cells expressing CD19 for 16 h. Statistics were performed between the calculated percentage of specific lysis of 2B4.ζ-CAR-SAP and the other CARs. A representative of three independent experiments is shown. (I) 2D12 cells non-transduced or transduced with 28.ζ-CAR, 2B4.ζ-CAR, or 2B4.ζ-CAR plus SAP were co-cultured with EL-4 cells expressing CD19. A representative of three independent experiments is shown. (J) T cells from C57BL/6 mice transduced with ζ-CAR, 28.ζ-CAR, 2B4.ζ-CAR, or 2B4.ζ-CAR plus SAP were cultured with IL-2 for 4 days. Apoptosis of each CAR-T cell (gated on GFP^+^) was assessed using annexin V/7-AAD double staining and FACS. A representative of two independent experiments is shown. Data are presented as mean values ± SD. Statistical analysis was performed using one-way ANOVA. ∗*p* < 0.05, ∗∗*p* < 0.01, ∗∗∗*p* < 0.001, ∗∗∗∗*p* < 0.0001. ns, not significant. See also Figure S4.

Next, we investigated whether 2B4.ζ-CAR functioned similarly to the original 2B4 by comparing it with 28.ζ- and ζ-CARs. We first performed a cytotoxicity assay by co-culture of primary CD8^+^ T cells expressing each CAR with tumor cells expressing CD19. As EL-4 cells are derived from thymic lymphoblasts, we additionally used a pre-B leukemia cell line known as BKO84 cells as the target cells (Figure S4C).^38^ As expected, no significant differences were observed in cytotoxicity against both EL-4 cells and BKO84 cells among 2B4.ζ-, ζ-, and 28.ζ-CAR-T cells (Figure 5A). The clustering composed of 2B4.ζ-CAR was stained densely by anti-pCD3ζ at the initiation of T cell–SLB contact, as well as the clustering composed of other CARs (Figure 5B). By quantification analysis, the proportion of cells forming pCD3ζ clusters and the ratio of anti-pCD3ζ staining to CAR-EGFP (pCD3ζ/CAR) demonstrated less significant differences among the three CARs (Figures 5C and 5D). Next, we introduced each CAR separately in CD4^+^ or CD8^+^ T cells and evaluated cytokine production by each CAR-T cell (Figure S4A). 2B4.ζ-CAR on CD4^+^ or CD8^+^ T cells had a lower capacity to produce IL-2, IFN-γ, and TNF-α than ζ- or 28.ζ-CAR when these CAR-T cells were stimulated with EL-4 cells expressing CD19 (Figure 5E). A similar trend was shown when those CAR-T cells were stimulated with CD19-expressing BKO84 cells (Figure 5F). Furthermore, 2B4.ζ-CAR-T cells produced less cytokine than T cells with a CAR incorporating the 4-1BB intracellular domain (BB.ζ-CAR) (Figure 5G).

In addition, we attempted to evaluate how the co-expression of SAP in 2B4.ζ-CAR-T cells modulated their function such as cytotoxicity against CD19-expressing target cells and cytokine production. 2B4.ζ-CAR-T cells introduced by SAP showed higher cytotoxicity and cytokine production than those without SAP introduction (Figures 5H and 5I). The phosphorylation state of Erk was restored by further introduction of SAP into 2B4.ζ-CAR-T cells (Figure S4H). Unfortunately, we found that co-expression of our 2B4.ζ-CAR vector and excessive SAP resulted in mild apoptosis in recipient primary T cells (Figure 5J); therefore, it would be difficult to expand and proliferate these engineered T cells expressing 2B4.ζ-CAR and SAP. This phenomenon is consistent with previous reports showing apoptotic responses elevated by SAP^14^^,^^39^^,^^40^; 2B4.ζ-CAR-T cells with SAP may be in an overactivated state. However, all data suggested that 2B4.ζ-CAR retained the original functional characteristics as well as the signaling pathway and that 2B4.ζ-CAR-T cells would be sufficient to function as a cytocidal tool for practical use without further expression of SAP.

### 2B4.ζ-chimeric antigen receptor-T cells acquire tumoricidal capacity without cytokine release *in vivo*

**Figure 6:**
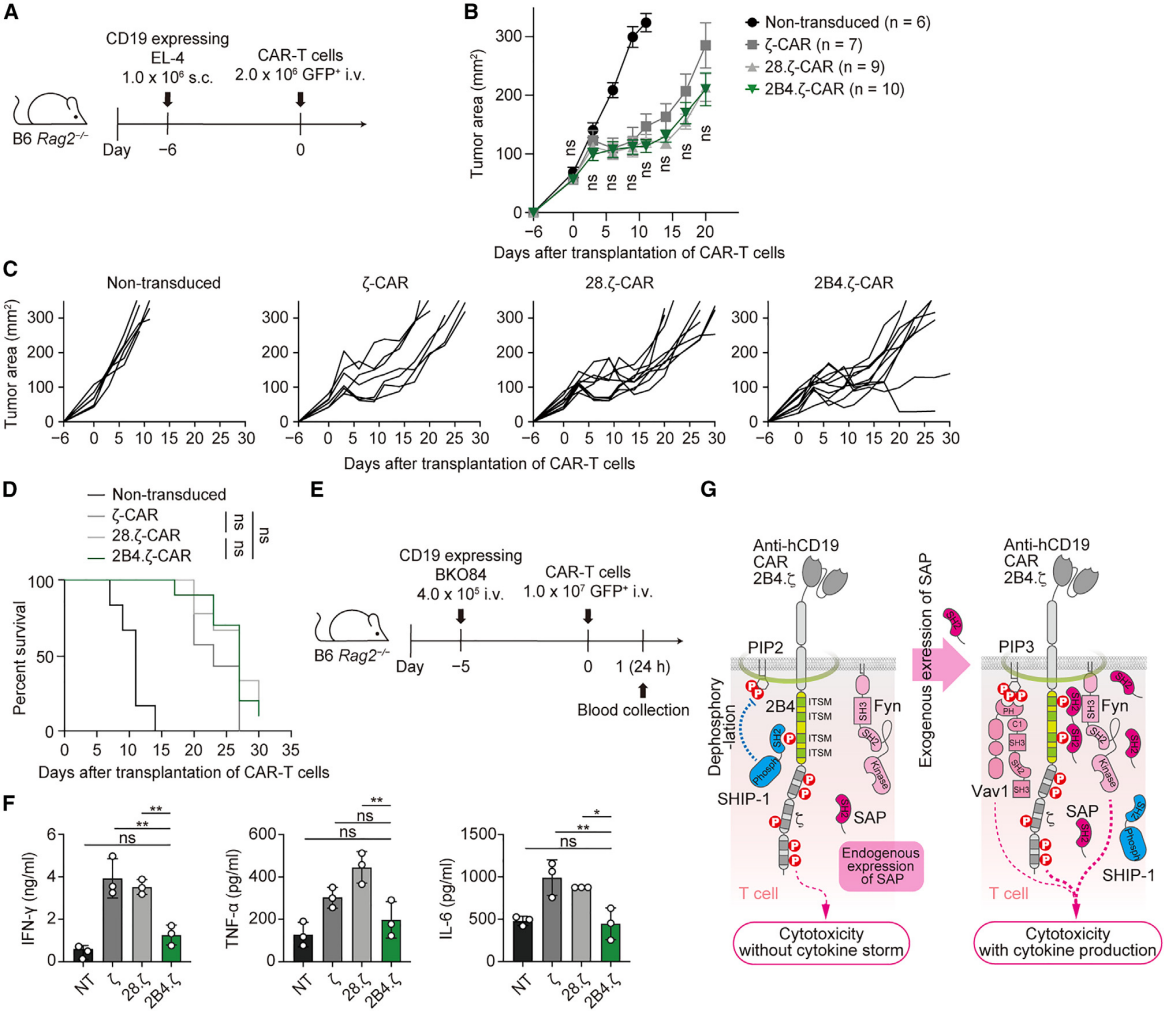
2B4.ζ-CAR-T cells show equivalent tumoricidal capacity and lower levels of inflammatory cytokines compared with other CAR-T cells *in vivo* (A) The schedule of CAR-T cell transfer to tumor-bearing mice *in vivo*. T cells from C57BL/6 mice non-transduced (*n* = 6) or transduced with ζ-CAR (*n* = 7), 28.ζ-CAR (*n* = 9), or h2B4.ζ-CAR (*n* = 10) were intravenously injected into B6 *Rag2*^*−/−*^ mice transplanted with EL-4 cells expressing CD19, 6 days before CAR-T cell transfer. (B) Tumor growth curves in each group. Statistics were performed among CAR groups. Mixed data from three independent experiments is shown. Bars, mean ± SEM. (C) Tumor growth curves of the individual mice. (D) Kaplan–Meier curves of percent survival in each group. (E) Schedule for *in vivo* study. T cells from C57BL/6 mice non-transduced or transduced with ζ-CAR, 28.ζ-CAR, or h2B4.ζ-CAR were intravenously injected into B6 *Rag2*^*−/−*^ mice transplanted with CD19^+^ BKO84 cells 5 days before CAR-T cell transfer. (F) Serum concentrations of IFN-γ, TNF-α, and IL-6 were measured 24 h after CAR-T cells injection by MILLIPLEX assay. A representative of two independent experiments is shown. (G) The schematic model of 2B4.ζ-CAR signaling pathways with little SAP in T cells (left) and with sufficient SAP in T cells (right). Data are presented as mean values ± SD. Statistical analysis was performed by one-way ANOVA or log rank test. ∗*p* < 0.05, ∗∗*p* < 0.01. ns, not significant.

Finally, we confirmed whether these *in vitro* findings with murine 2B4.ζ-CAR-T cells could be adapted to an *in vivo* tumor-bearing mouse model. To examine the cytotoxicity of the three types of CARs described above, we transplanted EL-4 tumor cells expressing CD19 into *Rag2*^*−/−*^ C57BL/6 mice subcutaneously (Figure 6A). Then, we intravenously injected each CAR-T cell into these tumor-bearing mice 6 days after the transplantation of the tumor cells and measured tumor sizes to compare their relative effects on tumor reduction. The 2B4.ζ-CAR-T cells demonstrated equivalent antitumor efficacy to the ζ- and 28.ζ-CAR-T cells (Figures 6B and 6C), and there was no significant difference in survival rates between the three groups (Figure 6D). We then intravenously administered CD19-expressing BKO84 cells to *Rag2*^*−/−*^ C57BL/6 mice to examine the amount of cytokine released after the administration of CAR-T cells (Figure 6E). Similar to the *in vitro* results, the serum concentrations of IFN-γ and TNF-α were significantly decreased in the 2B4.ζ-CAR group (Figure 6F). The 2B4.ζ-CAR group also demonstrated lower levels of IL-6, a critical cytokine associated with systemic inflammatory responses, compared with the other groups. Taken together, these *in vivo* results confirmed that 2B4.ζ-CAR-T cells, which retained the original signaling pathway of 2B4, had equivalent cytotoxic activity and simultaneously attenuated cytokine production compared with other types of CAR-T cells (Figure 6G).

## Discussion

In this study, we demonstrated the dynamics of signalosomes formed by 2B4 upon binding to its ligand, CD48, using a single-molecular imaging technique with an immunological synapse model to visualize the precise behavior of 2B4. Costimulatory signaling via 2B4 must have a complex mechanism due to its biphasic function, and research on signaling pathways in T cells has progressed less than that in NK cells. Our study may contribute to elucidating the controversial question of how 2B4 mediates T cell activity through 2B4–CD48 binding.

Using our imaging system, we found that 2B4 forms clusters with TCRs depending on 2B4–CD48 binding. This clustering of 2B4 could be identified as the minimal unit of 2B4 signaling, and constitutes a platform for receptor activation and signal propagation collaborating with TCR microclusters which are related to F-actin based projections.^41^^,^^42^ In the late phase of the T cell-SLB interaction, TCRs were dissociated from 2B4 to form a sole aggregate, which might be partly explained by the previous finding that some of the TCRs that accumulated at the center of immunological synapses were encased in extracellular vesicles.^43^^,^^44^ Even in the absence of TCR stimulation, 2B4 showed strong clustering in an antigen-independent manner through binding to CD48, which might be due to the effects of mouse CD2, another ligand of mouse CD48. CD2 is known to induce the autophosphorylation of Lck by binding to CD48 in mice or CD58 in humans, and it triggers weak TCR signals by forming a close contact at the adhesive surface.^45^^,^^46^^,^^47^ This interaction between CD2 and CD48 is reported to show weak phosphorylation of CD3ζ in the absence of TCR-MHC peptide binding. In our experiment for cytotoxicity, and in cytokine production assays, the baseline T cell response was a bit higher against CD48^+^ APCs than against CD48^−^ APCs. This finding is currently being investigated, and further research is needed to determine how binding of CD2 to CD48 or CD58 functions for T cell activation through cytoskeletal rearrangement.

We identified SHIP-1 as the molecule responsible for the inhibitory signal of 2B4 in T cells. Although 2B4 and other SLAM family receptors are known to preferentially associate with SHIP-1 in NK cells,^11^^,^^48^ to our knowledge, there are no reports demonstrating this association in T cells. The lipid phosphatase SHIP-1 modulates phosphoinositide 3-kinase signaling by degrading the signaling lipid PI(3,4,5)P_3_ to PI(3,4)P_2_, and it serves as a key scaffolding molecule in the formation of multi-protein complexes.^34^ In SAP-deficient NK cells, the increase in tyrosine phosphorylation of SHIP-1 was correlated with inhibition of downstream signaling via effector molecules such as phosphorylated Erk or Akt.^11^ Our study also confirmed the 2B4-mediated enhancement of phosphorylation of SHIP-1 and the attenuation of the phosphorylation of Erk, implying that a similar mechanism might work in T cells. In a previous report, SHIP-1 was shown to be responsible for most of the 2B4-mediated inhibition, with less than 25% of the inhibitory impact remaining in SHIP-1-deficient NK cells.^10^^,^^49^ Hence, SHIP-1 might mask the residual small inhibitory activities mediated by the other phosphatases such as SHP-1, SHP-2, and SHIP-2 (another member of the SHIP family). Although one report indicated the association of SHP-1 with 2B4 or SLAMF6 in T cells,^13^ our imaging system did not confirm the involvement of SHP-1. That article detected the recruitment of SHP-1 using SAP-deficient mice. Considering the previous research, the deletion of SHIP-1 or SAP might enhance and make apparent the behaviors of other phosphatases. Further analysis is required to clarify the involvement of not only SHIP-1 but also SHP-1 and SHP-2.

In this research, we demonstrated both a physical and functional connection of SAP with 2B4 and the positive effect of SAP on 2B4 signaling, showing the same trends as described in several reports: SAP activates SLAM family-mediated cellular activity in NK cells and T cells.^1^^,^^11^^,^^12^^,^^19^ Moreover, co-expression of SAP and SHIP-1 reduced the formation of SHIP-1 clusters, similar to when 2B4-4YF, instead of 2B4-WT, was introduced into T cells. This might be due to the overexpression of SAP occupying four ITSMs of the cytoplasmic domain of 2B4. Together, our results suggested that SAP acts on T cell activation, not only by leading to the Fyn–Vav1 pathway through direct recruitment to 2B4 but also by preventing the association of SHIP-1 with 2B4. Conversely, EAT-2, a member of the SAP family that acts as an adapter in NK-cell activation signaling,^35^^,^^36^^,^^37^ formed clusters with 2B4 but had no effect on cytokine production. As EAT-2 is rarely expressed endogenously in T cells, 2B4-mediated T cell activity seems to be little affected by EAT-2. SAP is also known to promote other steps of NK-cell activation, such as cytoskeletal reorganization and exocytosis and polarization of cytolytic granules, without connection to the SLAM family.^10^^,^^35^^,^^50^ We have clarified the spatiotemporal correlation between 2B4 and SAP, and our future challenge is to elucidate the various behaviors of SAP associated with the variety of functions mentioned above.

Biological outcomes from costimulatory signaling via SLAM family receptors, including 2B4, are debatable, and many studies have been reported under various settings. Some previous articles indicated that the upregulation of 2B4 expression might introduce T cell exhaustion.^6^^,^^51^^,^^52^ Genetic deletion of 2B4 has been shown to enhance the activation of the virus-specific T cell response in mice chronically infected with lymphocytic choriomeningitis virus.^53^ These studies demonstrated the inhibitory potential of 2B4 in T cells. Moreover, 2B4 upregulated by tuberculosis infection induced expression of *lnc-CD244*, a long non-coding RNA that suppresses the genes encoding IFN-γ and TNF-α and reduces cytokine production, consistent with the results of our study.^54^ Conversely, some reports imply the active function of 2B4 in CD8^+^ T cells, which enhances cytolytic activity and upregulates gene expression involved in effector function regulation.^5^^,^^55^ The expression levels of 2B4 and SAP should be considered when analyzing the biphasic properties of 2B4. In recent years, several reports have suggested that 2B4 acts on activation if NK cells express relatively higher amounts of SAP than 2B4, whereas it acts on inhibition if NK cells express lower amounts of SAP than 2B4 ^17,18^. Primary T cells examined in this experiment expressed little endogenous 2B4, but did express endogenous SAP to a certain extent. When 2B4 alone was transduced into these T cells, exogenous 2B4 might have led to the suppression of cytokine production by exceeding the expression of endogenous SAP. Meanwhile, the subsequent transduction of SAP changed the balance of their expression levels and promoted cytokine production. Although 2B4 signaling may be expressed in a variety of biological outputs through complex machineries and various situations; for example, expression levels of 2B4, its ligands, and other signaling molecules; type of cells; the surrounding tumor or inflammatory environments; T cell conditions; and other factors,^6^^,^^7^^,^^17^^,^^18^^,^^19^^,^^56^ it is clear that 2B4 plays an important role in regulating T cell functions and immunity.

We demonstrated that integrating the cytoplasmic domain of 2B4 into ζ-CAR preserves the unique signaling via 2B4. As a result, 2B4.ζ-CAR possessed the functional traits of 2B4, suppressing cytokine production while maintaining equivalent cytotoxic activity to other types of CARs. This might have an advantageous effect on CRS, a non-negligible adverse event of CAR-T cell therapy. In the pathogenesis of CRS, the first burst involves inflammatory cytokines such as IFN-γ, TNF-α, and GM-CSF released from CAR-T cells. This primary burst then over-activates macrophages and other immune cells, causing the secondary burst of cytokines such as IL-6 and IL-10, leading to CRS.^57^^,^^58^^,^^59^^,^^60^ In our *in vivo* experiments, 2B4.ζ-CAR decreased serum concentrations of IL-6, IFN-γ, and TNF-α, meaning that it attenuated the initial trigger of CRS. Tocilizumab, an IL-6 receptor antagonist, and other drugs, such as IL-1 blockers, TNF-α inhibitors, or anti-GM-CSF antibody, have been tested in this regard.^59^^,^^61^^,^^62^^,^^63^ However, these drugs have problems such as cost and drug-related side effects. As in our study, some research is underway to overcome CRS by adjusting the constructs of CAR or by modulating T cells.^64^^,^^65^^,^^66^ While several researchers have reported on 2B4-CAR-NK cells,^67^^,^^68^^,^^69^ only one previous article describes 2B4-CAR-T cells, showing that 2B4.ζ-CAR had superior tumor antigen-specific proliferation.^70^ 2B4.ζ-CAR provided potent costimulation for the tumor antigen-induced expansion of T cells undergoing phenotypic maturation toward CD8^+^ effector memory T cells. An earlier article about 2B4 demonstrated that 2B4 increased the proliferation of CD8^+^ T cells,^71^ suggesting that 2B4.ζ-CAR might reflect the original function of 2B4. Taken together, these results indicate that 2B4.ζ-CAR has the potential to attenuate cytokine toxicity (with reduced cost) and to excel in antigen-specific cell proliferation, as described above. Even though 2B4.ζ-CAR-T cells co-expressing SAP led to higher cytotoxicity and lower cytokine production than other types of CAR-T cells, they were difficult to establish due to the proapoptotic effect of SAP.^14^^,^^39^^,^^40^ Further studies are needed to better understand the underlying mechanisms and to create more advanced 2B4.ζ-CAR that exploits the inherent biphasic characteristics of 2B4 through optimal use of SAP.

In this article, we revealed the signaling mechanism of 2B4 in T cells utilizing an imaging system and confirmed that the features of 2B4 are commonly identified in 2B4.ζ-CAR, in which the intensity of SAP expression may be intentionally used to manipulate tumor-targeting cytotoxicity and proinflammatory cytokine production to an optimal degree. Our results suggest that 2B4.ζ-CAR has the potential to improve the next adoptive tumor immunotherapies.

### Limitations of the study

In our imaging system, because the behaviors of molecules were observed using T cells overexpressing each molecule, artifacts related to overexpression cannot be excluded. In addition, the cells primarily used in our imaging analysis were CD4^+^ T cells that expressed 2B4 at very low levels. For these reasons, it is important to note that the signalosome analysis in this study reflected not the normal physiology directly, but rather the molecular dynamics using an immunological synapse “model” from the scope of T cell engineering. The expression levels of each molecule are shown in the supplementary figures.

## Resource availability

### Lead contact

Further information and requests for resources and reagents should be directed to and will be fulfilled by the lead contact Prof. Tadashi Yokosuka (yokosuka@tokyo-med.ac.jp).

### Materials availability

All data supporting the conclusions included in the article are available from the lead contact upon request.

### Data and code availability

• Date: All data reported in the article are available from the lead contact upon request.
• Code: This article does not report the original code.
• All other requests: Any additional information required to reanalyze the data reported will be shared by the lead contact upon request.

## Acknowledgments

We thank Toshio Kitamura for the pMXs and pMCs retroviral vectors, Daisuke Kitamura for BKO84 cells, Masato Kubo for MAGPIX Dx analyzer, Malcolm K. Brenner for the CAR vector, and Mai Kozuka for secretarial assistance. This work was supported by JSPS KAKENHI (JP25113725, JP15H01194, JP16H06501, JP17H03600, JP19K22545, JP20H03536, JP23H02775, JP23H04790, T.Y.), PRESTO (U1114011, T.Y.) from Japan Science and Technology Agency, the Takeda Science Foundation (T.Y.), and the Naito Foundation (4465-135, T.Y.).

## Author contributions

R.M., E.W., W.N., and T.Y. designed the research; R.M, E.W., H.M., W.N., Y.Y., T.N., H.T., M.F., H.N., A.T., and T.Y. performed the research; R.M. and H.M. analyzed the data; E.W., M.S., and T.Y. supervised the research; and R.M. and T.Y. wrote the article.

## Declaration of interests

The authors declare no competing interests.

## STAR★Methods

### Key resources table

**Table:** 

REAGENT or RESOURCE	SOURCE	IDENTIFIER
**Antibodies**		

CD244.2 (m2B4 (B6) 458.1) PE	BioLegend	133507; RRID: AB_1626231
CD48 (HM48-1) PE	BioLegend	103405; RRID: AB_313020
human CD19 (HIB19) PE	BioLegend	302254; RRID: AB_2564142
human CD8α (SK1) APC	BioLegend	344722; RRID: AB_2075388
SH2D1A (SAP) (1A9)	BioLegend	690702; RRID: AB_2629824
Fyn (FYN-59)	BioLegend	626501; RRID: AB_2108785
I-A/I-E PE (M5/114.15.2)	BioLegend	107608; RRID: AB_313323
HRP-anti-rat IgG (polyclonal)	BioLegend	405405
isotype-matched control mouse IgG1 kappa PE	e-Bioscience	12-4714-82; RRID: AB_1944423
CD4 PE (RM4-5)	e-Bioscience	12-0042-82; RRID: AB_465510
CD8α PE (53-6.7)	e-Bioscience	15-0081-82; RRID: AB_468706
IL-2 purified (JES6-1A12)	e-Bioscience	14-7022-85; RRID: AB_468406
IL-2 biotin (JES6-5H4)	e-Bioscience	13-7021-85; RRID: AB_466899
Erk (polyclonal)	Cell Signaling Technology	4695S; RRID: AB_390779
Phospho Erk (polyclonal)	Cell Signaling Technology	4370S; RRID: AB_2315112
Rabbit IgG HRP	Cell Signaling Technology	7074; RRID: AB_2099233
Mouse IgG HRP	Cell Signaling Technology	7076; RRID: AB_330924
pY160 Vav (polyclonal)	Thermo Fisher Scientific	44-482; RRID: AB_2533661
Rat IgG (H+L) HRP	Thermo Fisher Scientific	31471; RRID: AB_10965062
Goat anti-Mouse IgG (H+L) Alexa Fluor647	Thermo Fisher Scientific	A-21235; RRID: AB_2535804
Goat anti-Rabbit IgG (H+L) Alexa Fluor647	Thermo Fisher Scientific	A-21244; RRID: AB_2535812
Goat anti-Rat IgG (H+L) Alexa Fluor647	Thermo Fisher Scientific	A-21247; RRID: AB_141778
HRP-anti-green fluorescent protein (GFP)	Miltenyi Biotec	130-091-833
biotin-labeled anti-human IgG, Fcγ	Jackson Immuno Research	109-065-098 ; RRID: AB_2337630
SHP-1 (C-19)	Santa Cruz	sc-287
SHP-2 (B-1)	Santa Cruz	sc-7384
SHIP-1 (P1C1)	Santa Cruz	sc-8425
Vav (D-7)	Santa Cruz	SC8039; RRID: AB_628428
Phospho tyrosine (4G10)	Merk Millipore	05-321; RRID: AB_310776
H-2K^b^ PE (AF6-88.5)	BD bioscience	561072; RRID: AB_394928
pCD3ζ Alexa Fluor647 (K25-407.69)	BD bioscience	558489
IFNγ biotin (XMG1.2)	BD bioscience	554410
IFNγ purified (R4-6A2)	BD bioscience	551216
CD48 (HM48-1)	Bio X cell	BE0147; RRID: AB_10949470
Human CD19 (4G7)	Bio X cell	BE0281; RRID: AB_2687804
CD28 (PV1)	Gift from R.Abe	N/A
CD3ζ (145-2c11)	Gift from J.Bluestone	N/A
TCRβ (H57-597)	Gift from RT.Kubo	N/A
I-Ek (14-4-4)	Gift from ML.Dustin	N/A
ICAM-1 (YN1/1.7.4)	Gift from ML.Dustin	N/A

**Bacterial and virus strains**		

DH5α	TOYOBO	DNA903

**Chemicals, peptides, and recombinant proteins**		

Mouse recombinant IL-2	Peprotech	212-12
MCC_88-103_ peptide	GenScript	N/A
OVA_257-264_ peptide	GenScript	N/A
Halo Tag STELLA Fluor 650 ligand	Promega	GCKA308-01
Halo Tag TMR ligand	Promega	G8252

**Critical commercial assays**		

Mojo Mouse CD4 T Cells Isolation Kit	BioLegend	480033
Mojo Mouse CD8 T Cells Isolation Kit	BioLegend	480035
APC Annexin V Apoptosis Detection Kit with 7-AAD	BioLegend	640930
TNF-α ELISA Kit	BioLegend	430901
CD19 MicroBeads, mouse	Miltenyi Biotec	130-121-301
DyLight 650 labeling Kit	Thermo Fisher Scieitific	84535
DyLight 549 labeling Kit	Thermo Fisher Scieitific	53044
MILLIPLEX MAP kits	Merk Millipore	48-602MAG
Fixation/ Permeabilization, solution Kit	BD bioscience	554714

**Experimental models: cell lines**		

Human: Platinum-E (Plat-E) Retroviral Packaging Cell Line	Gift from G.Nolan	N/A
Mouse: BKO84	Gift from D.Kitamura	N/A
Mouse: EL-4	ATCC	TIB-39; RRID: CVCL_0255
Hamster: BHK	ATCC	ACC-61, RRID:CVCL_1915
Mouse: AND TCR-transgenic T cell hybridoma (2D12)	Yokosuka et al., 2008	N/A
Mouse: OT-I TCR-transgenic T cell hybridoma (OT91)	This paper	N/A

**Experimental models: organisms/strains**		

Mouse: AND TCR-Tg/ *Rag2*^*-/-*^	Gift from R. Germian	N/A
Mouse: OT-I TCR-Tg/ *Rag2*^*-/-*^	Gift from W.Health	N/A
Mouse: *Rag2*^*-/-*^ Strain B6(Cg)-*Rag2*^*tm1.1Cgn*^/J	Gift from F.Alt	N/A
Mouse: C57BL/6	CLEA Japan	N/A
Mouse: B10.BR	Sankyo Labo	N/A

**Recombinant DNA**		

pMXs retroviral vector	Gift from T.Kitamura	N/A
pMCs retroviral vector	Gift from T.Kitamura	N/A
Renilla luciferase (RLuc) 8 fragment	Gift from Y.Okada	N/A
ζ-CAR fragment	Gift from MK.brenner	N/A
28.ζ-CAR fragment	Gift from MK.brenner	N/A

**Software and algorithms**		

Prism 7	GraphPad Software	https://www.graphpad.com/scientific-software/prism/
Flowjo v10	FlowJo	https://www.flowjo.com/solutions/flowjo
Adobe Illustrator	Adobe system	https://www.adobe.com/products/illustrator.html
ImageJ	NIH	https://imagej.nih.gov/ij/

### Experimental model and study participant details

#### Mice and cells

All animal experiments were performed in accordance with a protocol approved by the Animal Care and Use Committee of Tokyo Medical University (R3-009, R4-002, R5-059). Mice were maintained in specific-pathogen-free conditions with a 12 h/12 h light/dark cycle at 22°C and controlled humidity (60 ± 10%) at Tokyo Medical University. All experiments were performed on age- and sex-matched 6- to 12-week-old mice. Experimental and control animals were co-housed. Mice were humanely euthanized by cervical dislocation once they reached endpoints, such as reaching 300 mm^2^ in tumor area; loss of weight, mobility, or body condition; or having severe neurological disabilities.

The T-cell hybridoma expressing OT-I-TCR (OT91) was established by cell fusion of activated OT-I-TCR-Tg CD8^+^ T cells with TCR-negative lymphoma cell line, BW5147, as previously shown.^72^ The T-cell hybridoma expressing AND-TCR (2D12) was established by cell fusion of activated AND-TCR-Tg CD4^+^ T cells with BW5147.^73^ BKO84 was provided by D. Kitamura (Tokyo University of Science, Chiba, Japan).^38^ The EL-4 cell line was purchased from the American Type Culture Collection (ATCC). We deleted mCD48 (guides: CACCGACCATATAAACGTATCACC, AAACGGTGATACGTTTATATGGTC) from EL-4 cells using the CRISPR/Cas9 system (PX458, addgene, http://n2t.net/addgene:48138). All cells were confirmed to be free of mycoplasma contamination.

### Method details

#### Plasmid construction

EGFP-tagged m2B4, m2B4-4YF, mSHP-1, and mSHIP-1 were generated using the polymerase chain reaction (PCR) and subcloned into the retroviral vector pMXs (kindly provided by T. Kitamura, University of Tokyo, Tokyo, Japan).^74^ HaloTag-tagged mSAP, mEAT-2, mFyn, mSHP-1, mSHP-2, and mSHIP-1 were generated using PCR and subcloned into the pMXs retroviral vector. Mouse or human SAP was also subcloned into the pMXs-ires-hCD8α retroviral vector. hCD19, h2B4, and hSAP fragments were amplified from Jurkat cells, Raji cells, and a human NK cell line (NKL; kindly provided by H. Arase, Osaka University, Osaka, Japan).

The schematic diagrams of CARs are shown in Figure 4A. The fragments of ζ-CAR and 28.ζ-CAR, composed of anti-hCD19 antibody scFv (clone: FMC63), hinge of IgG4, CH3 domain of IgG1, transmembrane domain of hCD28, and cytoplasmic signaling domain of hCD28, h4-1BB, and/or hCD3ζ, were provided by M. K. Brenner (Baylor College of Medicine, Houston, TX, USA).^75^ EGFP-tagged ζ-CAR or 28.ζ-CAR genes were generated and subcloned into pMXs or pMCs (provided by T. Kitamura, University of Tokyo, Tokyo, Japan).^74^ We then integrated the cytoplasmic domains of m2B4 or h2B4 into ζ-CAR.

#### Primary cell culture and transduction

A packaging cell, PLAT-E, was transiently transduced with retroviral vectors using Lipofectamine 2000 (Invitrogen). The supernatants were concentrated 40- to 80-fold by centrifugation at 8,000 × *g* for 12 h. AND-TCR-Tg CD4^+^ T cells were purified from AND-TCR-Tg *Rag2*^*−*/*−*^ mice and stimulated with 5 μM MCC_88–103_ and irradiated spleen cells from B10.BR mice or with plate-bound anti-CD3ζ and anti-CD28 antibodies. OT-I-TCR-Tg CD8^+^ T cells were purified from OT-I-TCR-Tg *Rag2*^*−*/*−*^ mice and stimulated with 100 nM OVA_257–264_ and irradiated spleen cells from B6 mice, or with plate-bound anti-CD3ζ and anti-CD28 antibodies. One day after stimulation, the cells were suspended in retroviral supernatant with 10 μg/mL polybrene (Sigma-Aldrich) and 200 U/mL recombinant mouse IL-2 (Peprotech) and centrifuged at 1000 × *g* for 90 min at 37°C. On day 2, the cells were sorted to obtain populations with homogeneous fluorescence intensity, which were then maintained in RPMI 1640 medium (Sigma-Aldrich) containing 10% FCS (Thermo Fisher Scientific) and mouse IL-2.

#### Microscopy

Images were acquired using a confocal laser scanning microscope (TCS SP8, Leica Microsystems) comprising a 63× oil-immersion objective lens, high-sensitivity HyD detectors, and 488, 561, and 633 nm laser lines. LAS X software (Leica, Germany) was utilized for image acquisition. A TIRF analysis system was set up on a conventional inverted microscope (Ti-LAPP, Nikon, Tokyo, Japan) outfitted with a TIRF objective lens (Nikon), a scientific complementary metal oxide semiconductor (CMOS) camera (ORCA flash 4.0, Hamamatsu photonics), and fiber-coupled 488 nm lasers. The exposure time was set at 100 ms with a 2.5-s interval between time points. NIS-elements software (Nikon) was used for image acquisition. ImageJ software (National Institutes of Health [NIH], Bethesda, MD, USA, RRID:SCR_003070) was used for image processing and final figure preparation.

#### Planar bilayers

The purification and fluorescence labeling of GPI-anchored proteins were established according to published protocols.^76^ The mouse MHC class II molecule I-E^k^ with a GPI anchor (I-E^k^-GPI), the mouse MHC class I molecule H-2K^b^ with a GPI anchor (H-2K^b^-GPI), and mouse ICAM-1 with a GPI anchor (ICAM-1-GPI) were purified from transfected Chinese hamster ovary (CHO) and baby hamster kidney (BHK) cells, respectively, and were incorporated into dioleoyl phosphatidylcholine liposomes (Avanti Polar Lipids). BHK cells (ATCC) highly expressing mCD48-GPI and hCD19-GPI were established. mCD48-GPI and hCD19-GPI were purified from the lysates by affinity column with anti-mCD48 antibody (HM48-1, Bio X cell) and anti-hCD19 antibody (4G7, Bio X cell), respectively. The expression level of each GPI-anchored protein on the planar bilayer was quantified using silica beads with a diameter of 5 μm (Bangs Laboratories).^28^ The densities were calculated based on the standard beads, Quantum FITC-5 MESF (Bangs Laboratories), and adjusted to the approximate concentration by comparison with natural APCs: I-E^k^, 200 molecules/μm^2^; H-2K^b^, 200/μm^2^; mICAM-1, 150/μm^2^; mCD48, 150/μm^2^; and hCD19, 50/μm^2^ or 150/μm^2^. We prepared planar bilayers by mixing GPI-anchored proteins, dropping them on clean glass (40 mm glass coverslips, Bioptechs), and overlaying with a clean cover glass (Fisherbrand, Circles; Size: 12 mm) for 30 min. The planar bilayers were loaded with 10 μM MCC_88–103_ or 10 μM OVA_257–264_ in citrate buffer, pH 4.5, for 24 h at 37°C, blocked with 5% non-fat dried milk (Cell Signaling Technology) in phosphate-buffered saline (PBS) for 30 min at 37°C, cover glass removed, and left to stand in the assay medium (HEPES-buffered saline) containing 1% FCS, 2 mM MgCl_2_, and 1 mM CaCl_2_ in a flow cell chamber system (Bioptechs). The cells were prestained with DyLight 650-labeled anti-TCRβ (H57) Fab and/or HaloTag STELLA Fluor 650- or TMR-labeled ligands, then plated onto an SLB, and real-time imaged using confocal microscopy. For intracellular immunofluorescent staining, the cells on a planar bilayer were fixed with 4% paraformaldehyde for 10 min at room temperature, permeabilized with PBS containing 1% bovine serum albumin (staining buffer) and 0.05% Triton X-100 for 1 min, and stained with fluorescent-labeled anti-pCD3ζ for 30 min at room temperature. The images in the figures are representative data taken 2 or 10 min after contact.

#### Imaging processing and analysis

The size and fluorescence intensity of each region were analyzed in all images using ImageJ software. Fluorescence intensities were quantified based on the raw imaging data using the following formula: [intensity of fluorescence at each spot on a diagram] − [minimal intensity of each fluorescence on the entire line])/([mean intensity of each fluorescence on the entire line] − [minimal intensity of each fluorescence along the entire line].^28^ PCCs were subsequently calculated from each fold intensity. One PCC value was defined as the average value of the correlation coefficients of each microcluster on the two diagonal lines of one cell. The percentages of cells forming more than three microclusters 2 min after contact were presented as percent of T cells forming microclusters. All histograms in the figures show the fold fluorescence intensity of each molecule on the diagonal yellow lines in the differential interference contrast (DIC) images.

#### T cell–APC conjugation assay

mCD48-deleted or mCD48-expressing EL-4 cells transduced with I-E^k^ were prepulsed with 5 μM MCC_88–103_ or 100 nM OVA_257–264_ overnight at 37°C. m2B4-EGFP-expressing 2D12 cells or OT91 cells were cultured with those EL-4 cells. 2D12 cells expressing ζ-CAR or m2B4.ζ-CAR were cultured with EL-4 cells expressing hCD19. The real-time images were acquired 10 min after T cell–APC contacts using confocal microscopy.

#### Immunoprecipitation and western blotting

mCD48-deleted or mCD48-expressing EL-4 cells transduced with I-E^k^ were prepulsed with 5 μM MCC_88–103_ overnight at 37°C and washed before the assay. Next, 1 × 10^6^ 2D12 cells transduced with m2B4-EGFP, mSHIP-1-EGFP, or mSHP-1-EGFP were stimulated with 1 × 10^6^ EL-4 cells. Similarly, 1 × 10^6^ 2D12 cells transduced with m2B4.ζ-CAR were stimulated with 1 × 10^6^ EL-4 cells expressing hCD19. The cells were lysed with the lysis buffer (50 mM Tris-HCl, 50 mM NaCl, and 5 mM EDTA) containing 1% NP-40. Whole cell lysates (WCLs) or those immunoprecipitated by anti-GFP (RQ2, MBL International) were blotted with anti-GFP, anti-SHIP-1, anti-SHP-1, anti-SAP, anti-Fyn, anti-4G10, anti-Erk, anti-pErk, anti-Vav1, or anti-pVav1 as a first antibody, and HRP-anti-rabbit IgG polyclonal Abs, HRP-anti-mouse IgG polyclonal Abs, or HRP-anti-rat IgG polyclonal Abs as a second antibody. The intensity of each band was calculated using ImageJ software.

#### Flow cytometry

Cells were stained with antibodies for cell-surface molecules in Hanks’ balanced salt solution (HBSS). For intracellular staining with anti-SAP, anti-Fyn, anti-SHP-1, anti-SHP-2, and anti-SHIP-1, a fixation/permeabilization solution kit was used according to the manufacturer’s protocol. APC Annexin V Apoptosis Detection Kit with 7-AAD (BioLegend) was used for the apoptosis assay. A cell sorter, SH800S (Sony), was used for cell isolation, and cell analyzers, fluorescence-activated cell sorter (FACS) Canto II (BD), and Guava easyCyte (Merck Millipore) were used for analysis. Data were depicted using FlowJo software (Tree Star, Ashland, OR, USA).

#### *In vitro* cytokine assay

mCD48-deleted or mCD48-expressing EL-4 cells transduced with I-E^k^ were prepulsed with 10 μM MCC_88–103_ or 1 μM OVA_257–264_. Then, 5 × 10^4^ OT-I-TCR-Tg CD8^+^ T cells transduced with m2B4-EGFP were cultured with 5 × 10^4^ prepulsed EL-4 cells for 6 h. m2B4-EGFP-expressing 2 × 10^4^ 2D12 cells or OT91 cells not transduced or transduced with mSAP-IRES-hCD8α were cultured with 2 × 10^4^ prepulsed EL-4 cells for 16 h. Then, 1 × 10^5^ OT-I-TCR-Tg CD8^+^ T cells transduced with m2B4-EGFP were cultured with 5 × 10^5^ B6 splenocytes and 1 nM OVA_257–264_ for 6 h. A total of 5 × 10^4^ B6 CD8^+^ or CD4^+^ T cells transduced with each ζ-CAR, 28.ζ-CAR, or h2B4.ζ-CAR were cultured with the 5 × 10^4^ EL-4 cells or BKO84 cells expressing hCD19 for 6 h. The concentrations of IL-2, IFN-γ, and TNF-α were measured from the supernatant by enzyme-linked immunosorbent assay (ELISA). We used a TNF-α ELISA Kit (BioLegend) according to the manufacturer’s instructions. All experiments were performed in triplicate.

#### CTL killing assay

Renilla luciferase (RLuc) 8 fragment was amplified from Yellow Nano-lanterns (provided by Y. Okada, Riken, Japan)^77^ using PCR, and subcloned into the pMXs retroviral vector. mCD48-deleted or mCD48-expressing EL-4 cells transduced with RLuc8 were used as target cells and prepulsed with 1 μM OVA_257–264_. At the indicated E/T ratios, m2B4-EGFP-transduced OT-I-TCR-Tg CD8^+^ T cells were co-cultured with those EL-4 cells for 16 h. Similarly, B6 CD8^+^ T cells transduced with each ζ-CAR, 28.ζ-CAR, or h2B4.ζ-CAR were cultured with EL-4 cells or BKO84 cells transduced with hCD19 and RLuc8 for 16 h. After treatment with coelenterazine, an RLuc8 substrate (FUJIFILM Wako), the intensity of RLuc8 luminescence in live target cells was measured using a Lumino image analyzer, ImageQuant LAS4000 mini (GE Healthcare). All experiments were performed in triplicate.

#### Tumor-bearing mouse model

Here, 1 × 10^6^ EL-4 cells expressing hCD19 in 100 μL of PBS were inoculated subcutaneously in the dorsal region of 6- to 12-week-old female *Rag2*^−/−^ mice. Tumors were allowed to grow for 6 days before treatment, so that the tumor area reached between 5^2^ and 10^2^ mm^2^ at the time of CAR-T cell injection. Mice received 100 μL of PBS containing 2 × 10^6^ CD3^+^GFP^+^ζ-CAR-T cells, 28.ζ-CAR-T cells, h2B4.ζ-CAR-T cells, or untransduced T cells by intravenous injection in the tail vein. The tumor area was calculated using digital calipers as follows: (major axis of tumor) × (minor axis of tumor). For survival studies, the endpoint was established at a tumor area ≥300 mm^2^.

For measurement of cytokine levels in serum, 4 × 10^5^ BKO84 cells expressing hCD19 in 100 μL of PBS were inoculated intravenously into 6- to 12-week-old male *Rag2*^−/−^ mice via tail vein. Mice received 200 μL of PBS containing 1 × 10^7^ CD3^+^GFP^+^ζ-CAR-T cells, 28.ζ-CAR-T cells, h2B4.ζ-CAR-T cells, or untransduced T cells by intravenous injection in the tail vein. Peripheral blood was obtained from tail bleeding 24 h after the administration of CAR-T cells. The concentrations of IFN-γ, TNF-α, and IL-6 in serum were analyzed using MILLIPLEX MAP kits (Merck Millipore) and an analyzer, MAGPIX Dx (Luminex).

### Quantification and statistical analysis

Data are presented as the mean ± standard deviation (SD). Statistical analysis was performed using Student’s *t*-test, one-way analysis of variance (ANOVA), or log-rank test and GraphPad Prism software. *p*-values < 0.05 were considered to be statistically significant. As a measure of reproducibility, biological independent sample sizes and replicates are stated in each figure legend. ∗*p* < 0.05, ∗∗*p* < 0.01, ∗∗∗*p* < 0.001, ∗∗∗∗*p* < 0.0001.
