# Supplementary material for: Imaging of biphasic signalosomes constructed by checkpoint receptor 2B4 in conventional and chimeric antigen receptor-T cells
Source: iScience. 2024 Dec 21;28(1):111669. doi: 10.1016/j.isci.2024.111669 (PMC11780131; doi:10.1016/j.isci.2024.111669)
Supplement: Document S1. Figures S1–S4 [file mmc1.pdf]

iScience, Volume *28*

Supplemental information

Imaging of biphasic signalosomes constructed by checkpoint receptor 2B4 in conventional and chimeric antigen receptor-T cells

Ryohei Matsushima, Ei Wakamatsu, Hiroaki Machiyama, Wataru Nishi, Yosuke Yoshida, Tetsushi Nishikawa, Hiroko Toyota, Masae Furuhata, Hitoshi Nishijima, Arata Takeuchi, Makoto Suzuki, and Tadashi Yokosuka

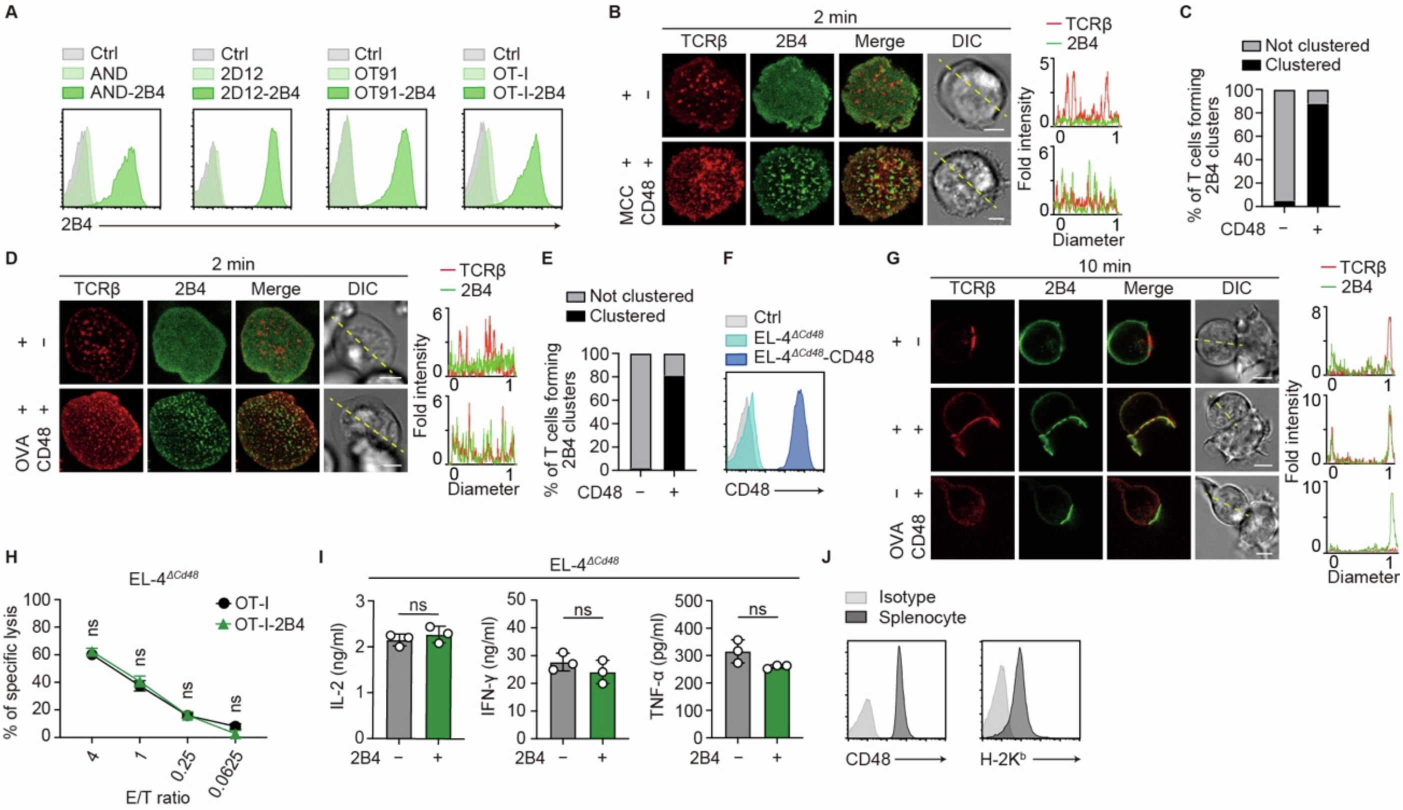

Figure S1. 2B4 forms microclusters in CD4^{+} or CD8^{+} T-cell hybridomas. Related to Figure 1. (**A**) FACS analysis of the expression of m2B4 on AND-TCR-Tg CD4^{+} T cells, 2D12 cells, OT91 cells, and OT-I-TCR-Tg CD8^{+} T cells non-transduced or transduced with m2B4-EGFP. (**B**) 2D12 cells transduced with m2B4-EGFP were plated onto an $MCC_{88–103}$-prepulsed SLB without or with mCD48-GPI. A representative of two independent experiments is shown. (**C**) Percentages of T cells forming 2B4 microclusters in (**B**) (n = 40). (**D**) OT91 cells transduced with m2B4-EGFP were plated onto an $OVA_{257–264}$-prepulsed SLB without or with mCD48-GPI. A representative of three independent experiments is shown. (**E**) Percentages of T cells forming 2B4 microclusters in (**D**) (n = 40). (**F**) FACS analysis of the expression of mCD48 on mCD48-deleted EL4 cells (EL-$4^{\Delta Cd48}$) and EL-$4^{\Delta Cd48}$ cells reconstituted by mCD48 (EL-$4^{\Delta Cd48}$-CD48). (**G**) OT91 cells expressing m2B4-EGFP were conjugated with mCD48-deleted or mCD48-expressing EL-4 cells prepulsed or not prepulsed by $OVA_{257–264}$. A representative of three independent experiments is shown. (**H**) The T cells in **Fig. 1F** were co-cultured with mCD48-deleted EL-4 (EL-$4^{\Delta Cd48}$) cells prepulsed by 1 µM $OVA_{257–264}$ at the indicated ratios for 16 h. A representative of three independent experiments is shown. (**I**) The T cells in **Fig. 1F** were co-cultured with EL-$4^{\Delta Cd48}$ cells prepulsed by 1 µM $OVA_{257–264}$. The concentrations of IL-2, IFN-γ, and TNF-α were measured by ELISA. A representative of three independent experiments is shown. (**J**) FACS analysis of the expression of mCD48 and H-2K^{b} on splenocytes prepared from C57BL/6 mice. Bars, 5 µm. Data are presented as mean values ± SD. Statistical analysis was performed by two-sided *t*-test. ns, not significant.

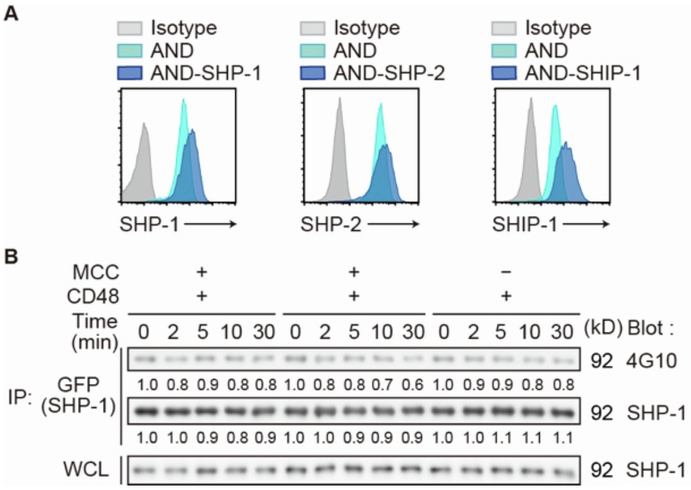

Figure S2. Expression levels of each phosphatase. Related to Figure 2. (**A**) FACS analysis of the expression of mSHP-1, mSHP-2, and mSHIP-1 in AND-TCR-Tg $CD4^{+}$ T cells non-transduced or transduced with those phosphatases. (**B**) 2D12 cells expressing EGFP-SHP-1 and 2B4-HaloTag were stimulated by mCD48-deleted or mCD48-expressing EL-4 cells not prepulsed or prepulsed by 10 μM $MCC_{88–103}$. Cells were lysed and immunoprecipitated for SHP-1 by anti-GFP. A representative of two independent experiments is shown.

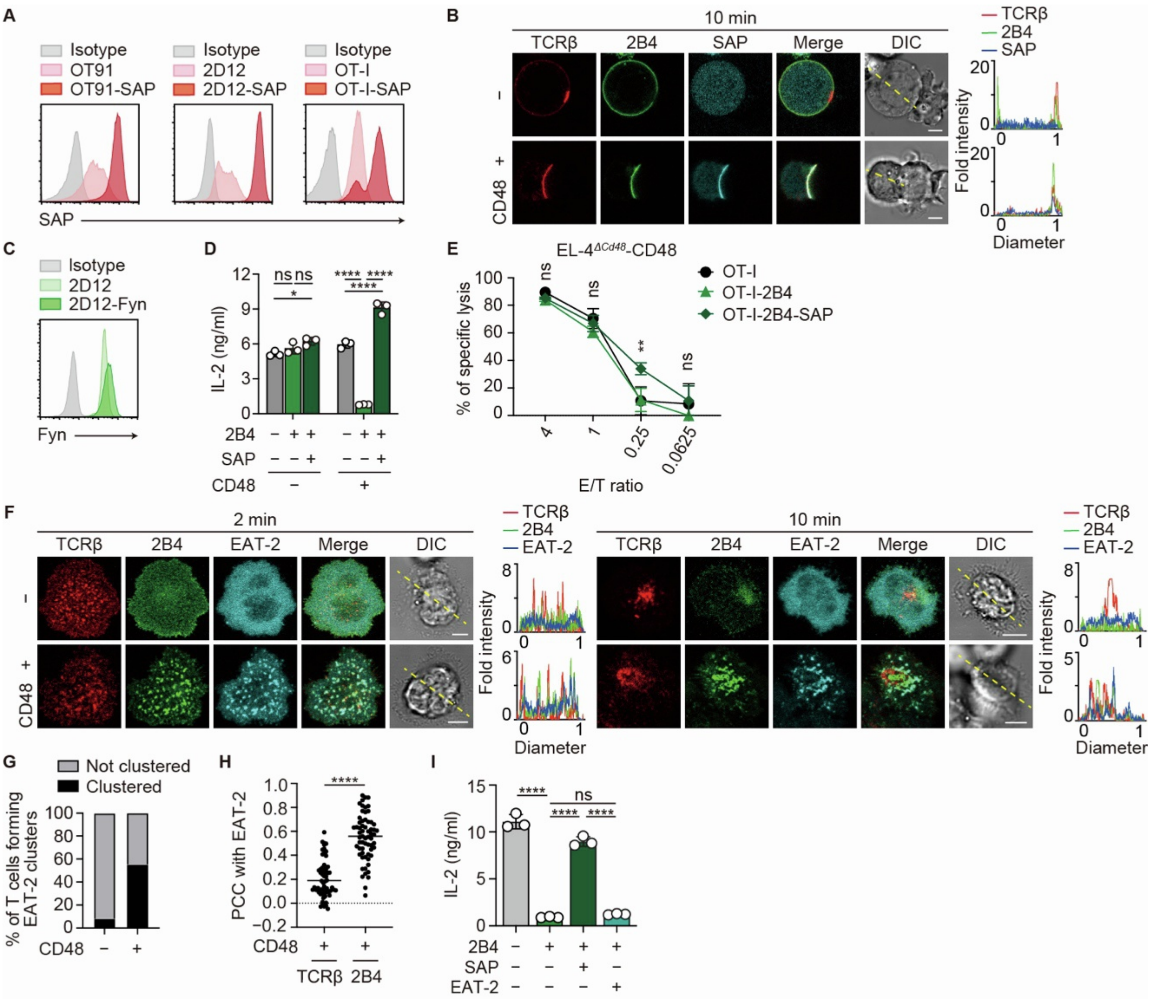

Figure S3. Involvement of SAP and EAT-2 in 2B4-mediated T-cell responses. Related to Figure 3. (**A**) FACS analysis of the expressions of mSAP in OT91 cells, 2D12 cells, and OT-I-TCR-Tg CD8^{+} T cells non-transduced or transduced with mSAP. (**B**) 2D12 cells expressing 2B4-EGFP and SAP-HaloTag were conjugated with CD48-deleted or CD48-expressing EL-4 cells prepulsed with MCC$_{88–103}$. A representative of two independent experiments is shown. (**C**) FACS analysis of the expression of mFyn in 2D12 cells non-transduced or transduced with mFyn. (**D**) 2D12 cells non-transduced or transduced with 2B4 alone or both 2B4 and SAP were co-cultured with mCD48-deleted or mCD48-expressing EL-4 cells prepulsed by 10 µM MCC$_{88–103}$. The concentration of IL-2 was measured by ELISA. A representative of three independent experiments is shown. (**E**) OT-I-TCR-Tg CD8^{+} T cells non-transduced or transduced with 2B4 alone or both 2B4 and SAP were co-cultured with CD48-expressing EL-4 (EL-4$^{\Delta Cd48}$-CD48) cells prepulsed by 1 µM OVA$_{257–264}$ for 16 h. Statistics were performed between the calculated percentage of specific lysis with or without SAP expression. A representative of two independent experiments is shown. (**F**) AND-TCR-Tg CD4^{+} T cells transduced with 2B4-EGFP and HaloTag-EAT-2 were plated onto an MCC$_{88–103}$-prepulsed SLB without or with CD48-GPI. A representative of two independent experiments is shown. (**G**) Percentages of T cells forming EAT-2 microclusters 2 min after contact in (**F**) (n = 50). (**H**) PCC was calculated between EAT-2/TCRβ (0.198 ± 0.16, n = 63) and EAT-2/2B4 (0.550 ± 0.19, n = 63) in T cells of the left bottom raw in (**F**). (**I**) OT91 cells non-transduced or transduced with 2B4 alone or both 2B4 and EAT-2 were co-cultured with mCD48-expressing EL-4 cells prepulsed by 1 µM OVA$_{257–264}$. The concentration of IL-2 was measured by ELISA. A representative of two independent experiments is shown. Bars, 5 µm. Data are presented as mean values ± SD. Statistical analysis was performed by two-sided *t*-test or one-way ANOVA. *$p < 0.05$, **$p < 0.01$, ****$p < 0.0001$. ns, not significant.

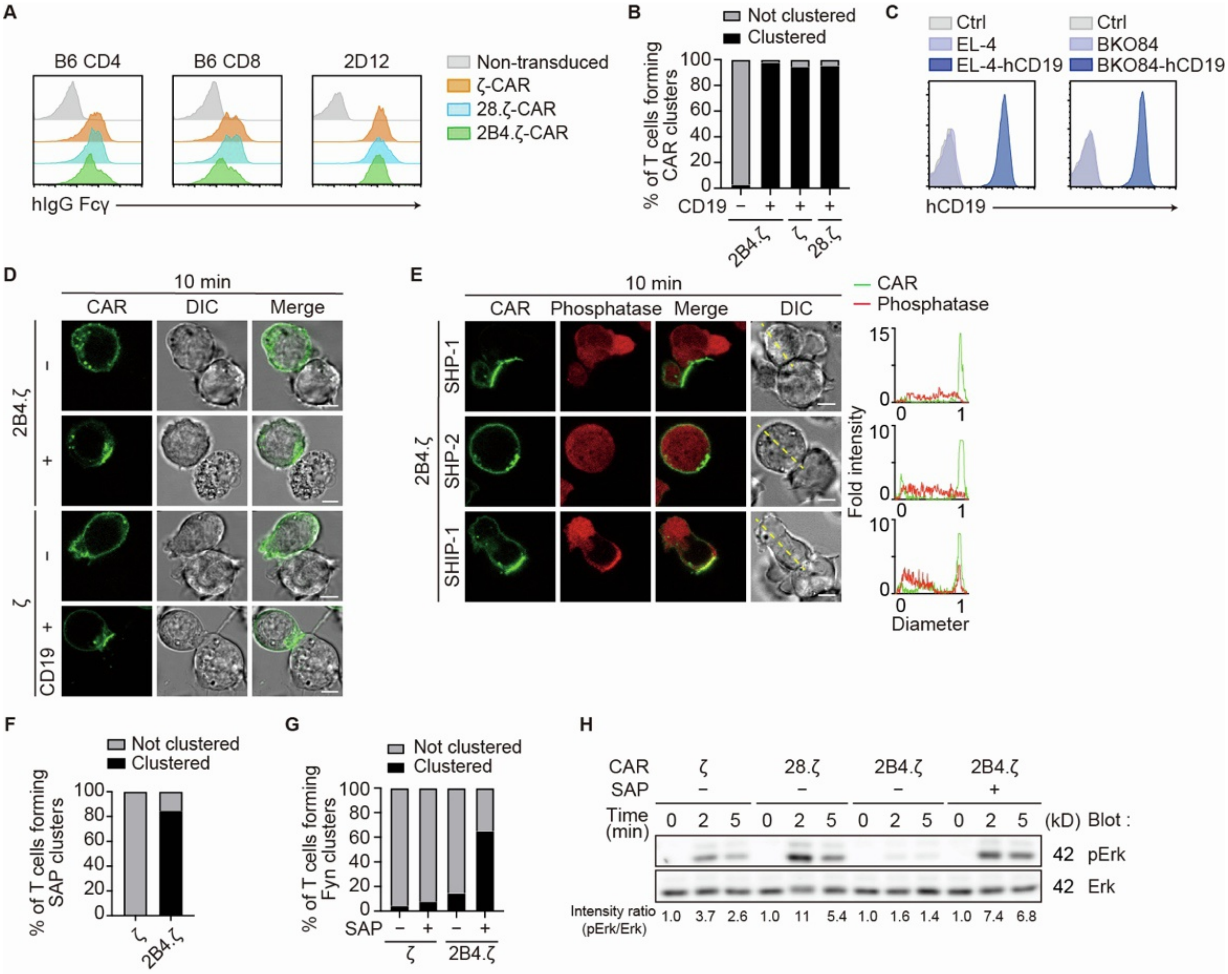

Figure S4. 2B4.ζ-CAR accumulates at a T cell–target cell interface and recruits SHIP-1, SAP, and Fyn. Related to Figure 4 and Figure 5. (**A**) FACS analysis of the cell-surface expression of CARs on CD4^{+} T cells, CD8^{+} T cells, and 2D12 cells non-transduced or transduced with ζ-CAR, 28.ζ-CAR, or 2B4.ζ-CAR. (**B**) Percentages of T cells forming CAR microclusters 2 min after contact in **Fig. 4D** (n = 30). (**C**) FACS analysis of the expressions of hCD19 on EL-4 cells or BKO84 cells non-transduced or transduced with hCD19. (**D**) 2D12 cells expressing 2B4.ζ-CAR-EGFP or ζ-CAR-EGFP were conjugated with EL-4 cells not expressing or expressing CD19. The real-time images were acquired 10 min after T cell–EL-4 cell contacts. A representative of three independent experiments is shown. (**E**) 2D12 cells expressing 2B4.ζ-CAR-EGFP and HaloTag-SHP-1, HaloTag-SHP-2, or SHIP-1-HaloTag were conjugated with EL-4 cells expressing CD19. A representative of two independent experiments is shown. (**F**) Percentages of T cells forming SAP microclusters in **Fig. 4H** (n = 50). (**G**) Percentages of T cells forming Fyn microclusters in **Fig. 4J** (n = 50). (**H**) 2D12 cells expressing ζ-CAR, 28.ζ-CAR, 2B4.ζ-CAR, or both 2B4.ζ-CAR and SAP were stimulated by EL-4 cells expressing CD19. The WCLs were blotted for pErk or Erk. A representative of three independent experiments is shown. Bars, 5 μm.
